# Synthesis, Structure and Electrochemical Properties of Acetamide- and Caprolactam-Containing Silicon Catecholates

**DOI:** 10.3390/molecules26123548

**Published:** 2021-06-10

**Authors:** Eugenia P. Kramarova, Alexander D. Volodin, Vadim V. Negrebetsky, Anastasia D. Shagina, Teimur M. Aliev, Pavel V. Dorovatovskii, Roman A. Novikov, Anna V. Vologzhanina, Alexander A. Korlyukov

**Affiliations:** 1 N.I. Pirogov Russian National Research Medical University, 1 Ostrovityanov St., 117997 Moscow, Russia; nikson1111@yahoo.com (E.P.K.); negrebetsky1@rsmu.ru (V.V.N.); alexkarry94@gmail.com (A.D.S.); 2 A.N. Nesmeyanov Institute of Organoelement Compounds, RAS. 28 Vavilova Str., 119991 Moscow, Russia; alex.d.volodin@gmail.com (A.D.V.); a.teimur1990@gmail.com (T.M.A.); vologzhanina@mail.ru (A.V.V.); 3 National Research Center “Kurchatov Institute”, 1 Acad. Kurchatov Sq., 123182 Moscow, Russia; paulgemini@mail.ru; 4 N.D. Zelinsky Institute of Organic Chemistry, RAS. 47 Leninsky Prosp., 119991 Moscow, Russia; novikovfff@bk.ru; 5 Engelhardt Institute of Molecular Biology, RAS. 32 Vavilov St., 119991 Moscow, Russia

**Keywords:** cyclic voltammetry, silicon catecholates, NMR studies, Si−C bond cleavage, X-ray diffraction

## Abstract

Hexacoordinated heteroligand silicon catecholates, although being prospective as easily soluble compounds with high hydrolytic stability and diverse redox properties, have been insufficiently studied. The transesterification of 1-(trimethoxysilylmethyl)-2-oxohexahydroaze or *N*-methyl-*N*-(trimethoxysilylmethyl)acetamide by two equivalents of catechol derivatives in the presence of dicyclohexylamine afforded a series of target compounds in good yield. The complexes were characterized using elemental analysis, FTIR, ^1^H, ^13^C and ^29^Si NMR spectra, X-ray crystallography and cyclic voltammetry. X-ray diffraction confirmed that the silicon atom possesses the octahedral geometry of the *SiCO_5_* polyhedron that remains unchanged in solution as it follows from ^29^Si NMR data. The compounds demonstrated up to three oxidation waves; and the reduction profile strongly depended on the nature of the substituents on a catecholate anion.

## 1. Introduction

Catecholate-containing silicon complexes are among the best-studied families of coordination compounds for this element. Starting from the synthesis of the anionic Si(Cat)_3_^2−^complex from SiO_2_, published in 1920, nearly 150 various compounds containing silicon atom connected with 1–3 catecholate ligands have been characterized (these can be found in the Cambridge Structural Database, released 2020.3). This interest is induced by the practical applications of silicon catecholates tuned by the electron-withdrawing properties of these anions. Their electron-withdrawing effect is strong enough, for example, to allow catecholate fragments of enterobacter to bind silicon even in conditions of living bacterial cell [1,2]. Silicon complexes with tetrahalogen-substituted catecholate ligands possess catalytic activity in the reactions of aldehyde hydrosilylation [3] related to the high Lewis acidity of Si(Cat)_2_ fragments that exceed the acidity of SbF_5_ [4,5]. Halogen-substituted catecholate anions are even able to stabilize a triplet state of silicon [6], which allows stable Si(Cat)_2_ radical to be obtained, with spin density distributed over the ligands. Some other substituted catecholates were used for the construction of macrocyclic and framework assemblies with large pores [1,2,7,8,9,10].

One of the research lines for silicon catecholate applications is devoted to the exploration of complexes with reversible electrochemical reduction and oxidation. It was demonstrated that pentacoordinated silicon atoms connected with an aryl or alkyl group beside two catecholate anions undergo irreversible electrochemical oxidation and can be used as a source of allyl radicals [11]. The binding of silicon benzoquinones with catecholate anions increased their stability towards electrochemical oxidation [12] that allowed a macrocyclic ligand with pentacoordinated silicon able to bind with various metal cations to be obtained. Silicon complexes containing both catecholate and 1,10-dipyridyl undergo reversible redox processes [13].

Among previously studied silicon complexes, the pentacoordinated ones are the most widespread; their number significantly exceeds that of the hexacoordinated. Among the hexacoordinated silicon complexes present in the CSD, the majority is represented by salts of Si(Cat)_3_ anions (for example see references. [14,15]), or complexes containing one catecholate anion only. Hexacoordinated silicon complexes containing two catecholate anions were typically obtained by the reaction of a Lewis base (for example, F^−^ or DME) with a Si(Cat)_2_ Lewis acid [4,5,16]. Thus, the number of anionic hexacoordinated heteroligand silicon complexes containing two catecholate ligands and an organic ligand coordinated through the covalent Si–C bond and an additional bond is limited by compounds containing an additional bond with amine [17,18] and with carbonyl groups [19]. Moreover, their redox behavior was not described. Previously, some of us synthesized and characterized two complexes containing acetamide and caprolactam ligands connected with a silicon atom beside two catecholate anions [19]. These complexes demonstrated high hydrolytic stability and moderate solubility with polar solvents (including water). We proposed that similar compounds can be prospective for the production of silicon complexes with reversible redox properties, as (i) their properties (solubility and redox potential) can be tuned by variation of the catecholate anions, (ii) additional coordination can increase the stability of complexes and allow at least partly reversible redox properties to be obtained. Besides, substituted catecholate anions should stabilize bis(catecholate)silicon radicals, obtained at the bond cleavage with acetamide or caprolactam fragments.

Herein we report the synthesis, IR,^1^H and ^13^C NMR spectra, crystal structures and redox properties of a series of anionic complexes containing two substituted catecholate anions, and acetamide or caprolactam. The nature of the substituents at the anions varies from donor to acceptor, which allows the analysis of the electronic effects on the coordination polyhedron of silicon atoms and the spectral and electrochemical properties of the complexes.

## 2. Results and Discussion

### 2.1. Synthesis

**Scheme 1:**
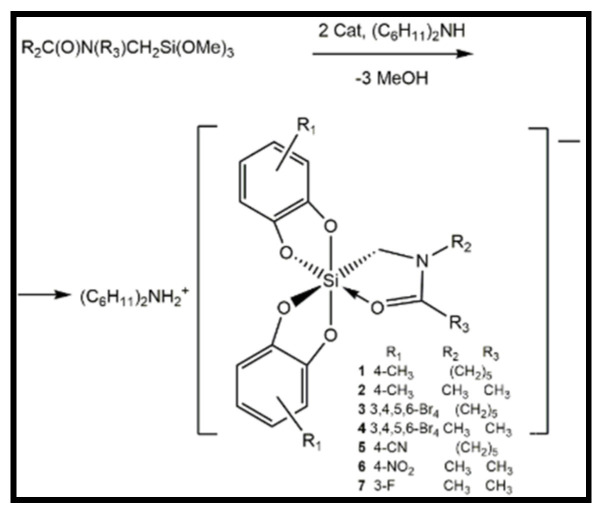
Synthetic route for compounds **1**–**7**.

*N*-Trimethoxysilylmethyl derivatives of *N*-methylacetamide and 2-oxohexahydroazepine were synthesized as described in reference [20]. Their transesterification by two equivalents of a series of commercially available catechol derivatives substituted by various electron donor and electron acceptor groups occurred on heating in o-xylene in the presence of one equivalent of dicyclohexylamine following the previously reported procedure [19]. The reaction afforded seven novel compounds listed in Scheme 1. The reaction yield varied from 78 to 91%, except for salt **4** (Yield is 47%).

The composition of salts **1**–**7** was confirmed using elemental analysis, IR spectroscopy, ^1^H, ^13^C and ^29^Si NMR spectra, as well as X-ray crystallography. The FTIR spectra (in solid KBr) exhibited the characteristic bands at 1602−1621 cm^−1^ and 1480–1529 cm^−1^ corresponding to stretching vibrations of, respectively, ν(C=O) and ν(C=N) groups involved in the five-membered chelate cycle. Bands at 1270–1220 cm^−1^ corresponded to Ph–O vibrations of catecholate anions. Thus, the elemental analysis, FTIR spectra and X-ray crystallography (see below) undoubtedly indicated that we had obtained heteroligand complexes containing hexacoordinated silicon atoms. The presence of non-equivalent ligands causes a prominent dipole moment of the anions. As result, the solids are soluble in polar solvents.

**Figure 1:**
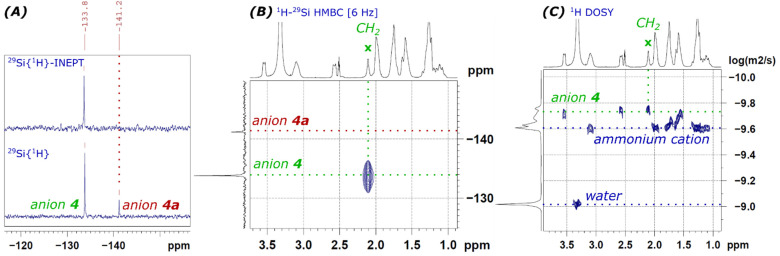
Crucial ^29^Si and diffusion NMR spectra for the elucidation of the [Si(C_6_O_2_Br_4_)_3_]^2−^ anion **4a**. (**A**) ^29^Si{^1^H} and ^29^Si-INEPT spectra in comparison. (**B)** Well-acquired ^1^H–^29^Si HMBC spectrum optimized on long-range *J* = 6 Hz. (**C**) ^1^H DOSY spectrum processed by the monoexponential fitting. All spectra were acquired at +50 °C in DMSO-*d*_6_ to prevent a slow rotational dynamic of sterically hindered molecules. In the ^29^Si-INEPT and ^1^H–^29^Si HMBC spectra, only RCH_2_[SiO_4_]^−^ was visible with the presence of protons near the silicon atom. In the ^1^H DOSY spectra, also only anion **4** was visible, in the form of two anionic and cationic parts with different diffusion coefficients. Proton free anion **4a** was invisible in all spectra except direct long acquisition of the ^29^Si (marked by red dot line), which was the best confirmation of its structure.

[Si^IV^*L*Cat^x^_2_]^−^ anions **1**–**7** (Cat^x^ = substituted catecholate ligand, L-bidentate chelate acetamide (**2**, **4**, **6**–**7**) or caprolactam (**1**, **3**, **5**) ligands) have a signal in ^29^Si NMR spectra in a very strong field at ca.−133 ppm, which is a typical chemical shift for pentacoordinated siloxane anions with formal excess of electron density on the silicon atom. The ^29^Si NMR spectrum of the [Si^IV^*L*Cat^x^_2_]^−^ anion **4** also has the second smallest signal in an even stronger field at –141 ppm, which was assigned to the anion **4a** [Si(C_6_O_2_Br_4_)_3_]^2−^ with hexacoordinated silicon atom and coordination polyhedron SiO_6_. To verify this hypothesis, we have carried out several special 2D NMR experiments (Figure 1). The main feature of this hexa-coordinated anion **4a** is a total absence of protons in the molecule, which leads to its invisibility in all spectra except direct long acquisition of the ^29^Si (Figure 1A). This is the best confirmation of anion structure. Thus, in the ^29^Si INEPT and ^1^H–^29^Si HMBC spectra, only [Si^IV^*L*Cat^x^_2_]^−^ **4** with the presence of protons near the silicon atom gave a strong signal/correlation peak (Figure 1A,B). In the ^1^H DOSY spectra, also only [Si^IV^*L*Cat^x^_2_]^−^ **4** is visible for the same reason (Figure 1C). Because it is an ionic compound, the anionic and cationic parts have different diffusion coefficients, which leads to the observation of two components in the diffusion spectra. The approximate ratio of compounds **4** and **4a** according to the results of ^29^Si NMR was 83:17.

Considering the reasons for the formation of compound **4a** [(C_6_H_11_)_2_NH_2_]_2_[Si(C_6_O_2_Br_4_)_3_]^2^, we assumed that it could be a decomposition product of compound **4**. In this case, *tris*-catecholates should be formed during the decomposition of other compounds studied in this work. After irradiation of compounds **2**, **6**, and **7** with a “white” UV radiation for 16 h, the signals of hexacoordinated silicon completely disappeared from their ^29^Si spectra. After appropriate irradiation of compound **4**, the signals did not completely disappear, but the change in intensity showed that the anion **4a** was more stable than the anion of compound **4** (the ratio of compounds **4**:**4a** changed to 74:26).

The peculiarities of the coordination environment and molecular structures of **1**–**7** in the solid state were studied with single-crystal X-ray diffraction.

### 2.2. Crystal Structures

**Figure 2:**
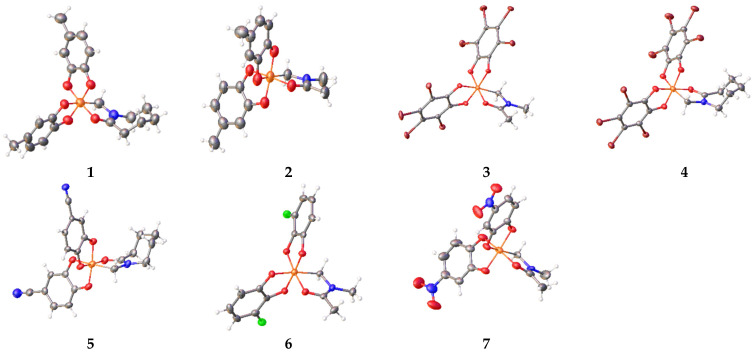
General view of. [Si^IV^*L*Cat^x^_2_]^–^ anions in **1**–**7** in representation of atoms with thermal ellipsoids (*p* = 50%). Hydrogen atoms are omitted. Color code: Br—brown; C—grey, F—green; N—blue; O—red; Si—orange.

Single-crystal X-ray diffraction confirmed that we had obtained a series of [Si^IV^*L*Cat^x^_2_]^−^ complexes with two bidentate chelate catecholate anions and a bidentate chelate acetamide (**2**, **4**, **6**–**7**) or caprolactam (**1**, **3**, **5**). Bis(cyclohexyl)ammonium (NH_2_[cyc]_2_^+^) acted as a counterion for all compounds. The asymmetric unit of **1** contained toluene molecules, and solid **2** also contained a 4-methyl-1,2-benzoquinone and solvent benzene molecules. In two isostructural crystal solvates of **3,** two symmetrically independent anions, and two types of cations, a potassium atom and NH_2_[cyc]_2_^+^ were found, as well as uncoordinated acetonitrile and water molecules. In Figure 2, the molecular view of the heteroligand anions in **1**–**7** is represented.

The silicon atom in these complexes possessed octahedral geometry. The Si–C and Si–O distances between the central atom and acetamide or caprolactam were the longest of the coordination bonds in the SiCO_5_ polyhedron (Table 1). The structure of the [Si^IV^*L*Cat^x^_2_]^−^anion closely resembled that of unsubstituted [Si^IV^*L*Cat_2_]^−^ [19]. The nature of substituents X almost did not affect the length of the coordination Si1−O1 bond (Table 1) with the carbonyl group of acetamide or caprolactam moieties. Indeed, the variation of the Si−O1 bond length was 0.03 Å only; and it was almost independent of the nature of R_1_, R_2_ and R_3_ substituents. At the same time, the length of Si−O bonds with a catecholate varied in a wider range (1.7638(17)–1.830(2) Å) that can be explained by either the effect of the Si–C bond, or by the participation of the corresponding oxygen atom in a strong hydrogen bond with NH_2_[cyc]_2_^+^ cations or non-coordinated Cat^X^ ligands, or by coordination with potassium cation (in **6** and **7**). Thus, [Si^IV^*L*Cat^x^_2_]^−^ proved to be a rigid, very stable cage system almost insensitive to the influence of the nature of a ligand and intermolecular interactions.

**Figure 3:**
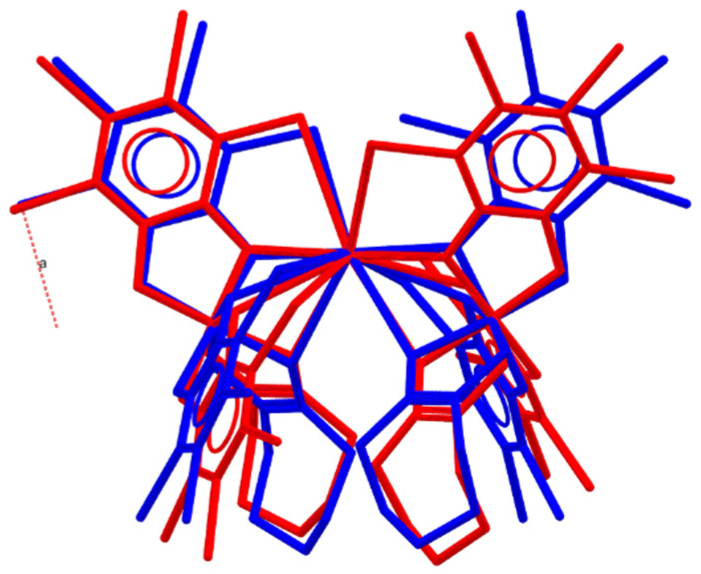
{K[Si*L*Cat^x^_2_]_2_}^−^ anions represented with overlayed K and Si atoms in solid **3a** and **3b**.

Salt **3** is the only one of the studied compounds that contains potassium ions besides (NH_2_[cyc]_2_^+^) ions. This complex was recrystallized from acetonitrile dried upon KOH. This solvent probably became the source of potassium ions upon recrystallization, as elemental analysis of the powder sample taken from the reaction mixture did not show the presence of the alkali ion. Two different single crystals taken from the reaction mixture were isostructural but contained various sets of solvent molecules. Potassium cations in solid **3a** and **3b** coordinated two silicon-containing anions. All 3,4,5,6-tetrabromocatecolate anions were coordinated by the potassium atom through Br and O atoms forming a five-membered cycle (r(K-Br) = 3.587(4)–3.730(5)Å, r(K-O) = 2.73(1)–2.80(1) Å), and caprolactam was coordinated through an oxygen atom (r(K-O) = 2.85(1)–2.93(1)Å). The molecular geometries of the trinuclear {K[Si*L*Cat^x^_2_]_2_}^−^ anions were similar (Figure 3).

One of the most noticeable structural features of the crystal structure was the packing motifs formed by the R_2_CHR_3_NCOSi(cat^X^) anions. In previously studied structures, the R_1_CHR_2_NCOSi(Cat) and N[cyc]_2_^+^ cations formed chains via N−H…O bonds. Besides, non-coordinated catechol was also found to be H–bonded to anions. In solid **1**−**7,** the cation (NH_2_[cyc]_2_^+^) formed two N−H…O bonds with catecholate anions or solvent molecules. In crystals of **1**−**3a** and **5,** water molecules or catechol co-formers also took part in H–bonding. However, neither acetamide nor caprolactam took part in H–bonding in all these compounds. Note, that H–bonds weaken the Si−O_Cat_ bond involved in the H–bonding, as was demonstrated by the DFT calculations [19]. This effect can be demonstrated in the example of solvent-free salt **7**. The Si1−O2 and Si1−O3 bonds involved in N–H…O bonding with the cation were elongated as compared with the Si1−O4 and Si1−O5 bonds that took part in weaker C–H…O interactions (1.805(2) and 1.809(2) Å as compared with 1.768(2) and 1.789(2) Å, Table 1). In our opinion, these data indicate, that in water or other solutions containing H-bond donors, the catecholate anions will be the main goal of solvent “attack”. More details about supramolecular organization can be found in Supplementary Materials.

### 2.3. Electrochemical Characterization

To date, the mechanism of electrochemical oxidation/reduction of hypervalent silicon compounds is still not revealed completely. In literature, there are few examples of electrochemical oxidation/reduction of compounds of silicon with derivatives of pyrocatechol [11,12,13]. As it follows from quantum chemical calculations, there should be several high lying redox-active orbitals localized on catecholate ligands [12]. The influence of organic substituents on the oxidation potential can be explained by π→σ* donation from respective orbitals of catecholate ligands to those of a substituent. In the case of pentacoordinated bis(catecholato)silicon compounds with different exocyclic substituents R [11] it was found that the oxidation potential varied from +0.28 to +0.89 V, for R = NH_2_ and Ph, respectively (DMSO, Fc^+^/Fc). The oxidation processes for the latter compounds were the irreversible and homolytic cleavage of the Si−R bond. In macrocyclic compound [(XbicH_2_)SiPh][HCl_2_] [12] the value of irreversible oxidation potential was almost the same as in the abovementioned bis(catecholato)silicon complex with R = Ph (+0.81, Fc^+^/Fc, CH_2_Cl_2_). In general, the coordination of the metal atoms to oxygen ones of catecholate ligands leads to a significant decrease of oxidation potentials. We expected that transition from pentacoordinated to hexacoordinated silicon would significantly change the oxidation potential or would make the oxidation/reduction process reversible because an additional coordination bond led to the increase of steric overcrowding and rearrangement of electron density. The role of hypervalent bonding with the carbonyl group of L ligand is unclear. The latter is often described as the n→σ* interaction, however, two types of donation, from a lone electron pair of oxygen atoms of catecholate ligand to the lowest unoccupied orbital of acetamide (caprolactame) substituent and in the reverse direction. In addition, the energies of redox-active orbitals depend on the nature of the substituents in benzene cycles of catecholate ligands. As a result, the oxidation potentials could be increased or decreased, and the values of the oxidation potentials cannot be easily predicted.

**Figure 4:**
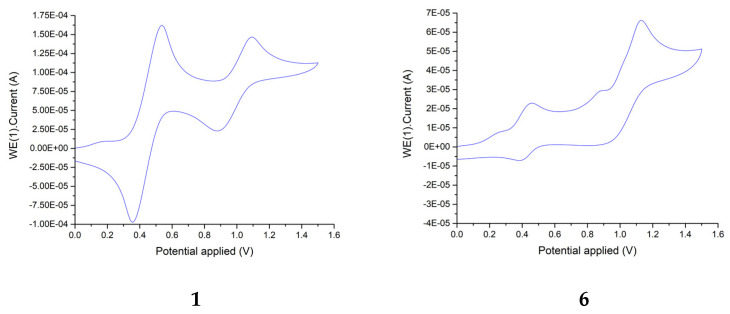
CV plots for compounds **1** (caprolactam ligand and donor Me substituent in catecholate fragments) and **6** (acetamide moiety and acceptor NO_2_ substituents in catecholate fragments).

To determine the oxidation potentials of compounds, cyclic voltammetry was carried out. Clean voltammograms were obtained showing irreversible oxidation waves (ESI). The oxidation potentials were determined to be between +0.006 V for 5 to +0.928 V versus Fc/Fc^+^ pair in DMSO for **7**. It was shown that the oxidation potentials were apparently dependent on the nature of the substituent in the phenyl cycle (Figure 4). Caprolactam-containing compounds with Me and CN substituents demonstrated the lowest potentials, so all three oxidation waves can be revealed upon measurements in DMSO. In acetamide-containing anions, going from electron donor Me group (**2**, E1_ox_ = 0.649 V) to electron acceptor NO_2_ (**6**, E1_ox_ = 0.593 V) and F (**7**, E1_ox_ = 0.312 V) one could see a decrease in the oxidation potential value (Table 2). In the case of acceptor substituents, only one or two potentials were detected upon the measurements in DMSO. Possibly, the second and the third potentials were too high to measure in the above-mentioned conditions. Unfortunately, the measurements in water carried out for **4** (the other compounds were insoluble in distilled water or DMF) in water showed the presence of only one wide oxidation wave. Analogously to catecholate complexes with pentacoordinated silicon atoms, the first oxidation potentials in most of the compounds can be attributed to the homolytic cleavage of Si−C bond with the acetamide or caprolactam moiety.

## 3. Materials and Methods

### 3.1. General

All reagents and solvents were purchased from SigmaAldrich (Merck KGaA, Darmstadt, Germany). The IR spectra were recorded on a «Bruker Tensor-27» ( Bruker Optik GmbH, Ettlingen, Germany) instrument in KBr pellets. The NMR spectra were measured on Bruker Avance II spectrometer (^1^H, 400 MHz; ^13^C, 75 MHz, (Bruker BioSpin, Silberstreifen, Germany) in CD_3_CN, DMSO-d_6_, CDCl_3_ relative to Me_4_Si as the internal standard. ^1^H, ^13^C, ^29^Si, ^29^Si INEPT, 2D ^1^H–^29^Si HMBC, and 2D ^1^H DOSY NMR spectra were recorded using a Bruker Avance-III 400MHz (Bruker BioSpin, Silberstreifen, Germany) and a Bruker Avance-III-HD 300 MHz NMR spectrometers (Bruker BioSpin, Silberstreifen, Germany) in DMSO-*d*_6_ at +30 °C and +50 °C, using standard modern pulse sequences with a z-gradient, if necessary, from Bruker library and using a Bruker Topspin 3.2 and 3.5 software for spectra processing; chemical shifts for all nuclei are auto-referenced precisely by spectrometers using a deuterium lock-channel for DMSO-*d*_6_^2^H signal (ca. 2.50 ppm). Elemental analysis was carried out in the Laboratory of Organic Microanalysis of INEOS RAS, using Carlo-Erba CE-1106 element analyzer (Carlo-Erba Strumentazione, Milan, Italy). Melting temperatures were measured with Stuart SMP10.

### 3.2. Synthesis

*N*-(Trimethoxysilylmethy)-*N*-methylacetamide and *N*-(trimethoxysilylmethyl)-*N*-hexahydrozepin-2-one were synthesized as described in reference [20]. Pyrocatechol derivatives and dicyclohexylamine were purchased from Aldrich.

Dicyclohexylammonium *bis*-(4-methyl-1,2-catechelato-κ^2^*O,O′*)-[(2-oxo-1-hexahydroazepin-2-yl)-methyl-κ^2^*C,O*]silicone(**IV**) (**1**). The mixture of 1-(trimethoxysilylmethyl)-2-oxohexahydroazepine (1.23 g, 5 mmol), of 4-methylcatechol (1.24 g, 10 mmol) and dicyclohexylamine (0.9 g, 5 mmol) was dissolved in 15 mL o-xylene at stirring and heated at 130–140 °C for 2 h. After cooling to room temperature, crystals were formed over 10 h. The solid was recrystallized from toluene resulting in a pure product (2.25 g, 78% yield), m.p. 178–180 °C. IR-spectra (KBr, ν, cm^−1^): 1602 (NCO), 1497, 1447 (NH_2_^+^bend.), 1251 (Ar-O). ^1^H NMR (400 MHz, DMSO-*d*_6_) δ: 1.00–2.02 (m, 30H: 20H from C_6_H_11_ and 10H from lactam), 2.05 (s, 3H, PhCH_3_), 2.11 (s, 3H, PhCH_3_), 2.54 (s, 1H, SiCH_2_N), 3.02 (m, 2H, NCH), 3.47 (s, 1H, SiCH_2_N), 5.97–6.62 (m, 6H, Ar), 8.68 (s, 2H, NH_2_). ^13^C{^1^H} NMR (101 MHz, DMSO-*d*_6_) δ:20.41, 21.25, 22.27, 24.01, 24.95, 25.93, 29.08, 29.36, 31.47, 46.51, 51.52, 52.18, 108.32, 110.38, 115.18, 115.46, 116.41, 119.49, 123.39, 127.90, 142.88, 145.01, 149.74, 152.02, 176.09.^29^Si NMR (60MHz, DMSO-*d*_6_) δ: −132.01. HRMS calcd. (*m*/*z*) for C_21_H_24_NO_5_Si: 398.1429; found: 398.1438.

Dicyclohexylammonium *bis*-(4-methyl-1,2-catechelato-κ^2^*O,O′*)-[*N*-methyl-*N*-(acetamido)-methyl-κ^2^*C,O*]silicone(**IV**) (**2**). The mixture of 4-methylcatechol (0.6 g, 5 mmol), of *N*-methyl-*N*-(trimethoxysilylmethyl)acetamide (0.5 g, 2.5 mmol), and dicyclohexylamine (0.45 g, 2.5 mmol) was dissolved in o-xylene (20 mL) and stirred and heated at 130–140 °C for 1 h. After cooling to room temperature, the mixture was extracted with hexane (50 and 30 mL). Then, the crystalline material was formed (1.07 g, 79% yield), m.p. 118–121 °C. Single crystals were given by recrystallization from benzene. IR-spectra (KBr pellets, ν, cm^−1^): 1618, (NCO),1495, 1450 (NH_2_^+^bend.), 1251 (Ar−O). ^1^H NMR (400 MHz, DMSO-*d*_6_) δ:0.96–2.00 (m, 20H, C_6_H_11_), 2.06 (s, 6H,CH_3_CO and PhCH_3_), 2.11 (s, 3H, PhCH_3_),2.54 (s, 1H, SiCH_2_N), 2.80 (s, 1H, SiCH_2_N), 3.04 (m, 2H, NCH), 3.04 (s, 3H, NCH_3_), 5.96–6.65 (m, 6H, Ar), 8.62 (s, 2H, NH_2_). ^13^C NMR (101 MHz, DMSO-*d*_6_) δ:16.91, 20.41, 21.24, 24.00, 24.92, 29.19, 38.18, 45.49, 52.20, 108.37, 110.42, 115.26, 115.46, 116.41, 119.49, 123.48, 127.90, 142.89, 145.02, 149.58, 151.86, 171.22. ^29^Si NMR (60MHz, DMSO-d_6_)δ: −133.18. HRMS calcd. (*m*/*z*) for C_18_H_20_NO_5_Si: 358.1116; found: 358.1112.

Dicyclohexylammonium potassium bis(bis-(3,4,5,6-tetrabromo-1,2-catechelato- κ^2^*O,O′*)-[(2-oxo-1-hexahydroazepin-2-yl)-methyl-κ^2^*C,O*]silicone(**IV**)) (**3**). The synthesis was carried out as described (2.60 g, 88% yield), m.p. 210–214 °C. Single crystals were given by recrystallization from acetonitrile. IR-spectra (ν, cm^−1^): 1611, 1529 (NCO), 1446 (NH_2_^+^bend.), 1221 (Ar−O). ^1^H NMR (400 MHz, DMSO-*d*_6_) δ: 0.97–1.97(m, 30H: 20H from C_6_H_11_ and 10H from lactam), 2.44 (s, 1H, SiCH_2_N), 3.04 (m, 2H, NCH), 3.46 (s, 1H, SiCH_2_N), 8.05 (s, 2H, NH_2_). ^13^C NMR (101 MHz, DMSO-*d*_6_) δ: 21.87, 23.92, 24.81, 25.24, 28.95, 29.11, 30.74, 44.86, 51.32, 52.22, 104.78, 111.36, 150.13, 177.43. ^29^Si NMR (60 MHz, DMSO-*d*_6_) δ: −141.19, −133.78. HRMS calcd. (*m*/*z*, max peak) for C_16_H_8_Br_8_NO_5_Si: 961.3564; found: 961.3559.

Dicyclohexylammonium *bis*-(3,4,5,6-tetrabromo-1,2-catechelato-κ^2^*O,O′*)-[*N*-methyl-*N*-(acetamido)-methyl-κ^2^*C,O*]silicone(**IV**) (**4**). The synthesis was carried out as described (1.35 g, 47% yield), m.p. 225–230 °C. A suitable for X-ray diffraction single crystal was obtained without further purification. IR-spectra (ν, cm^−1^): 1621 (NCO), 1580 (Ar), 1448 (NH_2_^+^bend.), 1273, 1220 (Ar−O). ^1^H NMR (400 MHz, DMSO-*d*_6_) δ:0.96–2.03(m,20H, C_6_H_11_), 2.17 (s, 3H,CH_3_CO), 2.54 (s, 1H, SiCH_2_N), 3.10 (m, 2H, NCH), 3.13 (s, 3H, NCH_3_), 3.31 (s, 1H, SiCH_2_N), 8.17 (s, 2H, NH_2_). ^13^C NMR (101 MHz, DMSO-*d*_6_) δ:16.51, 23.90, 24.80, 28.86, 38.17, 44.33, 52.18, 105.21, 111.62, 149.98, 172.36.^29^Si NMR (60MHz, DMSO-*d*_6_) δ: −135.23. HRMS calcd. (*m*/*z*, max peak) for C_19_H_12_Br_8_NO_5_Si: 1001.3877; found: 1001.3874.

Dicyclohexylammonium *bis*-(4-nitrilo-1,2-catechelato-κ^2^*O,O′*)-[(2-oxo-1-hexahydroazepin-2-yl)-methyl-κ^2^*C,O*] silicone (**IV**) (**5**). The synthesis was carried out as described with recalculation to 5 mmol (2.57 g, 85% yield), 128–131 °C. A suitable for X-ray diffraction single crystal was obtained without further purification. IR-spectra (ν, cm^–1^): 2208 (CN), 1608 (NCO). ^1^H NMR (400 MHz, DMSO-*d*_6_) δ: 0.97–2.00 (m,30H: 20H from C_6_H_11_ and 10H from lactam), 2.54 (s, 1H, SiCH_2_N), 2.99 (m, 2H, NCH), 3.50 (m, 1H, SiCH_2_N), 6.37 (d, *J* = 7.9 Hz,2H, Ar), 6.53 (d, *J* = 1.8 Hz,2H, Ar), 6.79 (dm, *J* = 8.0 Hz,2H, Ar).^13^C{^1^H} NMR (101 MHz, DMSO-*d*_6_) δ:22.26, 24.17, 25.11, 25.82, 29.09, 29.68, 31.24, 45.97, 51.62, 52.27, 96.84, 96.89, 110.26, 111.09, 116.32, 117.90, 120.13, 121.71, 123.27, 124.98, 146.50, 151.88, 152.12, 157.25, 177.06.^29^Si NMR (60MHz, DMSO-*d*_6_)δ: −132.95. HRMScalcd. (*m*/*z*) for C_21_H_18_N_3_O_5_Si: 420.1021; found: 420.1004.

Dicyclohexylammonium *bis*-(4-nitro-1,2-catechelato-κ^2^*O,O′*)-[*N*-methyl-*N*-(acetamido)-methyl-κ^2^*C,O*]silicone (**IV**) (**6**).The synthesis was carried out as described with recalculation to 2.9 mmol (1.58 g, 91% yield), m.p. 179–180 °C. A suitable for X-ray diffraction single crystal was obtained without further purification. IR-spectra (ν, cm^–1^): 1613 (NCO), 1584 (Ar), 1490 (NH_2_^+^bend.), 1262, 1251 (Ar−O). ^1^H NMR (400 MHz, DMSO-*d*_6_) δ:0.97–2.06 (m, 20H, C_6_H_11_), 2.13(s, 3H, C(O)CH_3_), 2.73(s, 1H, SiCH_2_N), 3.08 (m, 2H, NCH), 3.10 (s, 3H, NCH_3_), 3.55 (s, 1H, SiCH_2_N), 6.42 (d, *J* = 8.5 Hz, 2H, Ar),7.08 (d, *J* = 2.6 Hz,2H, Ar),7.49 (dm, *J* = 8.5 Hz, 2H, Ar).^13^C{^1^H} NMR (101 MHz, DMSO-*d*_6_)δ:16.71, 24.04, 24.94, 29.04, 38.24, 44.84, 52.29, 103.87, 108.75, 116.38, 137.60, 137.67, 151.43, 160.25, 172.46.^29^Si NMR (60MHz, DMSO-*d*_6_)δ: −132.46. HRMS calcd. (*m*/*z*) for C_16_H_14_N_3_O_9_Si: 420.0505; found: 420.0477.

Dicyclohexylammonium *bis*-(3-fluoro-1,2-catechelato-κ^2^*O,O′*)-[*N*-methyl-*N*-(acetamido)-methyl-κ^2^*C,O*]silicone (**IV**) (**7**). The synthesis was carried out as described with recalculation to 3.5 mmol (1.66 g, 86% yield), m.p. 223−225 °C. A suitable for X-ray diffraction single crystal was obtained without further purification. IR-spectra (ν, cm^–1^): 1612 (NCO), 1588 (Ar), 1487 (NH_2_^+^bend.), 1271 (Ar−O). ^1^H NMR (400 MHz, DMSO-*d*_6_) δ: 0.98–2.02 (m, 20H, C_6_H_11_), 2.12(s, 3H, C(O)CH_3_), 2.55(d, *J* = 3.9 Hz,1H, SiCH_2_N), 3.07 (m, 2H, NCH), 3.10 (s, 3H, NCH_3_), 3.31 (d, *J* = 4.1 Hz,1H, SiCH_2_N), 6.06–6.29 (m,6H, Ar).^13^C{^1^H} NMR (101 MHz, DMSO-*d*_6_)δ:16.73, 23.93, 24.82, 28.99, 38.13, 45.18, 52.34, 103.82 (d, *J* =18.7 Hz), 105.91, 114.27 (d, *J* =8.7 Hz),138.23 (d, *J* = 10.2 Hz), 148.56 (d, *J* = 236 Hz), 154.44 (d, *J* = 8.1 Hz), 171.65.^29^Si NMR (60MHz, DMSO-*d*_6_)δ: −132.41. HRMS calcd. (*m*/*z*) for C_16_H_14_F_2_NO_5_Si: 366.0615; found: 366.0603.

### 3.3. X-ray Diffraction Studies

Single crystal X-ray studies of **4**–**7** were carried out in the Center for Molecule Composition Studies of INEOS RAS, using MoKα radiation at 120 K. X-ray datasets for **1**–**3** were collected in Kurchatov Centre for Synchrotron Radiation and Nanotechnology using the “Belok” beamline ( λ = 0.79313 Å, T = 100 K) because of the low diffraction quality of crystals of **1** and **2** and high absorption of **3**. The structures were solved by by dual-space algorithm starting from Patterson superposition minimum function method and refined in anisotropic approximation for non-hydrogen atoms. Hydrogen atoms of methyl, methylene and aromatic fragments were calculated according to the ideal geometry and refined with constraints applied to C–H bond lengths and equivalent displacement parameters (U_eq_(H) = 1.2U_eq_(X), X-central atom of XH₂ group; U_eq_(H) = 1.5U_eq_(Y) for methyl groups and water molecules). Solvated molecules of C_6_H_6_, toluene and molecules of non-coordinated ligands in **1** and **2** are disordered. The geometry of those moieties was refined using rigid body approximation for aromatic benzene rings. In the case of **7** fluorine atoms in Cat^x^ ligands were disordered over two positions in ratio 1:5. An attempt to refine C−F distances without restraints led to unrealistic values, while the usage of DFIX restraints caused a noticeable increase in R-values. All structures were solved with the ShelXT [21] program and refined with the ShelXL [22] program. Molecular graphics were drawn using OLEX2 [23] program. The structures **3a** and **3b** were refined as inversion twins, using TWIN and BASF instructions (Flack parameters were equal to 0.095(8) and 0.368(15), respectively). Crystal parameters and refinement details are listed in Table 3. CCDC 2035549-2035556 contain the supplementary crystallographic data for **1**–**7**. These data can be obtained free of charge from The Cambridge Crystallographic Data Centre via https://www.ccdc.cam.ac.uk/structures.

### 3.4. Electrochemistry

Electrochemical analysis was conducted on an Autolab PGSTAT128N Potentiostat-Galvanostat with NOVA 2.0 software, using a typical three-electrode array. Platinum working electrode was used, as well as Ag/AgCl (saturated KCl solution) as a reference electrode, and platinum wire as a counter electrode. A 0.1M TBAPF_6_ solution in DMSO was used as the supporting electrolyte. All scans were performed with 100 mV/s scan rate. Before each experiment, the solution was purged with N_2_ gas for 5 min and the working electrode was thoroughly polished. All the tests were carried out at room temperature. The measured potentials were determined versus Fc/Fc^+^ pair.

## 4. Conclusions

In conclusion, a series of heteroligand silicon catecholates was successfully synthesized with satisfactory yields, using a transesterification reaction. The protocol can be easily modified to obtain other anionic complexes of hexacoordinated silicon based on various catechol derivatives and *N*-methylamides. This synthetic development is important for the development of hydrolytically stable silicon compounds with enhanced solubility for processing in electrochemical reactions. The only exclusion is anion complex **4** where the cleavage of Si–C bond was detected under synthetic conditions or UV irradiation. For the acetamide-containing series we found correlation between the first oxidation potential and the electronic properties of a substituent at catechol anion. The more negative charge the substituent has, the lower the oxidation potential of the complex. Caprolactam-containing compounds demonstrated the lowest potentials, so that up to three oxidation waves can be revealed for these compounds. Thus, the electrochemical properties of this family of compounds can be easily tuned by changing the ligands, and the search for the representatives able to exhibit reversible redox processes is underway now.

## Figures and Tables

**Table 1:** Selected geometrical parameters (Å, º) of *SiCO_5_* polyhedra in **1**–**7**.

	1	2	3a ^1^	3b ^1^	4	5	6	7
Si1-O1	1.877(2)	1.8851(15)	1.880(12)	1.896(6)	1.867(3)	1.882(3)	1.860(17)	1.8873(19)
Si1-O2	1.788(2)	1.7935(14)	1.792(12)	1.777(6)	1.784(3)	1.809(3)	1.778(2)	1.8090(18)
Si1-O3	1.800(2)	1.7638(17)	1.795(11)	1.795(6)	1.830(3)	1.782(3)	1.7861(18)	1.8052(19)
Si1-O4	1.761(2)	1.7889(17)	1.798(11)	1.795(6)	1.784(3)	1.798(4)	1.7868(18)	1.7894(18)
Si1-O5	1.801(2)	1.7934(16)	1.801(11)	1.807(6)	1.815(3)	1.815(3)	1.8036(17)	1.768(2)
Si1-C1	1.918(3)	1.923(2)	1.927(19)	1.918(8)	1.925(4)	1.932(4)	1.996(17)	1.923(3)
O1Si1O2	174.71(11)	174.95(8)	176.5(6)	177.5(3)	175.38(14)	175.97(14)	174.0(5)	173.13(9)
O3Si1O4	173.31(11)	174.13(8)	172.8(6)	173.7(3)	175.10(14)	173.59(14)	176.33(9)	175.12(10)
O5Si1C1	171.91(12)	170.34(8)	167.2(7)	168.4(3)	169.15(16)	172.13(15)	169.6(5)	172.07(10)

^1^ For salt **3,** two solvates were characterized using X-ray diffraction.

**Table 2:** Cyclic voltammogram data of **1**–**7** recorded in DMSO, 0.1 M TBAPF6 as supporting electrolyte, Ag/AgCl in saturated KCl solution reference electrode, Pt working electrode. C(comp) = 1 mM.

Compound	Fc	E1_ox_ (V)	E2_ox_ (V)	E3_ox_ (V)
**1**	0.487	0.006	0.346	0.497
**2**	0.447	0.649		
**3**	0.464	0.869		
**4**	0.459	0.654	0.928	
**5**	0.485	0.047	0.511	0.845
**6**	0.486	0.593	0.837	
**7**	0.489	0.312	0.756	

**Table 3:** Crystal parameters and refinement details for salts **1**–**7**.

	1∙C_7_H_8_O_2_∙2C_7_H_8_	2∙C_7_H_8_O_2_∙2.5C_6_H_6_	3a∙H_2_O∙0.5C_2_H_3_N	3b∙2C_2_H_3_N	4	5∙2H_2_O	6	7
Formula	C_54_H_72_N_2_O_7_Si	C_52_H_67_N_2_O_7_Si	C_51_H_51·50_Br_16_KN_3·50_O_11_Si_2_	C_54_H_54_Br_16_KN_5_O_10_Si_2_	C_28_H_32_Br_8_N_2_O_5_Si	C_33_H_46_N_4_O_7_Si	C_28_H_38_N_4_O_9_Si	C_28_H_38_F_2_N_2_O_5_Si
Fw	889.22	860.16	2263.29	2306.86	1143.92	638.83	602.71	548.69
Crystal system	Monoclinic	Monoclinic	Monoclinic	Monoclinic	Monoclinic	Orthorhombic	Triclinic	Monoclinic
Space group	P2_1_/n	P2_1_/c	Cc	Cc	P2_1_	Pbca	P1̅	P2_1_/c
a, Å	16.231(3)	12.302(3)	20.160(4)	20.754(4)	9.3422(5)	15.651(16)	11.2726(17)	9.192(2)
b, Å	15.185(3)	15.613(3)	20.620(4)	20.697(4)	20.4930(11)	19.49(2)	11.7569(17)	20.522(6)
c, Å	19.933(4)	25.066(5)	17.620(4)	18.740(4)	9.4596(5)	21.76(3)	13.4405(19)	14.798(3)
α, °	90	90	90	90	90	90	73.242(3)	90
β, °	92.22(3)	100.08(3)	113.78(3)	115.59(3)	98.6190(10)	90	80.444(4)	105.325(10)
γ, °	90	90	90	90	90	90	77.905(4)	90
V, Å	4909.2(17)	4740.1(17)	6703(3)	7260(3)	1790.58(17)	6637(13)	1657.1(4)	2692.2(12)
Z	4	4	4	4	2	8	2	4
d_calc_, g cm^−1^	1.203	1.205	2.243	2.111	2.122	1.279	1.208	1.354
μ, mm^−1^	0.132	0.134	12.912	11.924	9.031	0.123	0.124	0.143
F(000)	1920	1852	4308	4400	1096	2736	640	1168
Ref. coll.	29,344	43,014	46,282	40,319	40,981	23,440	16,813	18,206
Ref. ind. R_int_	10,704 0.086	10,824 0.052	14,922 0.039	16,243 0.046	10,879 0.038	7593 0.145	9841 0.062	8030 (0.099)
Ref. obs. (I > 2σ(I))	5451	6465	13,780	11,537	9735	3465	4296	3840
Parameters	717	576	780	831	399	424	413	363
R_1_ (I > 2σ(I))	0.067	0.057	0.033	0.066	0.025	0.073	0.068	0.066
wR_2_ (all refls.)	0.205	0.171	0.087	0.204	0.050	0.196	0.172	0.171
GOF	1.013	1.038	1.039	1.038	0.993	1.001	0.929	0.958
*ρ*ₘᵢₙ/*ρ*ₘₐₓ, eÅ^−3^	−0.276/0.253	−0.322/0.242	−1.028/0.946	−0.717/1.565	−0.494/0.776	−0.298/0.463	−0.288/0.303	−0.616/0.623
Flack	-	-	0.095(8)	0.368(15)	0.006(4)	-	-	-
No. of restr.	19	79	46	128	1	0	2	6

## Data Availability

All spectra and XRD data are available from the authors.

## Supplementary Materials

The following are available online. Figures S1–S10: H-bonded architectures in crystal structure of **1**–**5**, Figures S11–S16: CV plots of **1**–**7**, Figures S17–S44: ^1^H, ^13^C, ^29^Si NMR and HRMS spectra of **1**–**7**.

## Sample Availability

Samples of the compounds **1**–**7** are available from the authors.
