# Supplementary material for: Synthesis, Structure and Electrochemical Properties of Acetamide- and Caprolactam-Containing Silicon Catecholates
Source: Molecules. 2021 Jun 10;26(12):3548. doi: 10.3390/molecules26123548 (PMC8230554; doi:10.3390/molecules26123548)

molecules

MDPI

Synthesis, Structure and Electrochemical Properties of Acetamide- and Caprolactam-Containing Silicon Catecholates

Eugenia P. Kramarova [1], Alexander D. Volodin [2], Vadim V. Negrebetsky [1], Anastasia D. Shagina [1], Teimur M. Aliev [2], Pavel V. Dorovatovskii [3], Roman A. Novikov [4,5], Anna V. Vologzhanina [2] and Alexander A. Korlyukov [1,2,*]

[1] N.I. Pirogov Russian National Research Medical University. 1 Ostrovityanov St., 117997 Moscow, Russia; nikson1111@yahoo.com (E.P.K.); negrebetsky1@rsmu.ru (V.V.N.); alexkarry94@gmail.com (A.D.S.)
[2] A.N. Nesmeyanov Institute of Organoelement Compounds, RAS. 28 Vavilova str., 119991 Moscow, Russia; alex.d.volodin@gmail.com (A.D.V.); a.teimur1990@gmail.com (T.M.A.); vologzhanina@mail.ru (A.V.V.)
[3] National Research Center "Kurchatov Institute". 1 Acad. Kurchatov Sq., 123182 Moscow, Russia; paulgemini@mail.ru
[4] N.D. Zelinsky Institute of Organic Chemistry, RAS. 47 Leninsky prosp., 119991 Moscow, Russia; novikovfff@bk.ru
[5] Engelhardt Institute of Molecular Biology, RAS. 32 Vavilov st., 119991 Moscow, Russia
* Correspondence: alex@xrlab.ineos.ac.ru; Tel.: 007(495)1359214

Supramolecular organization in crystals of 1–8

The similar packing motif is observed for compounds **1** and **2** (Figure 1s and 2S) where noncoordinated 4-methyl catecholate ligand form N-H…O and O-H…O bonded chains. The crystal structure of **7** does not contain non-coordinated ligand, however, anions and $N[cyc]_2^+$ cations are also assembled into N-H…O bonded chains (Figure 3S). Unlike to **1**, **2** and **7** in crystal of **4** cations and anions formed H-bonded dimers and supramolecular structure is maintained owing to weak Br…π and H…Br interactions (Figure 4S). The presence of nitro group in **6** caused the change of dimer structure as compared to **4**. In this case, the centrosymmetrical tetramers assembled by four N-H…O bonds with oxygen atoms of nitrogroup and 3-nitro catecholate ligands are formed (Figure 5S).

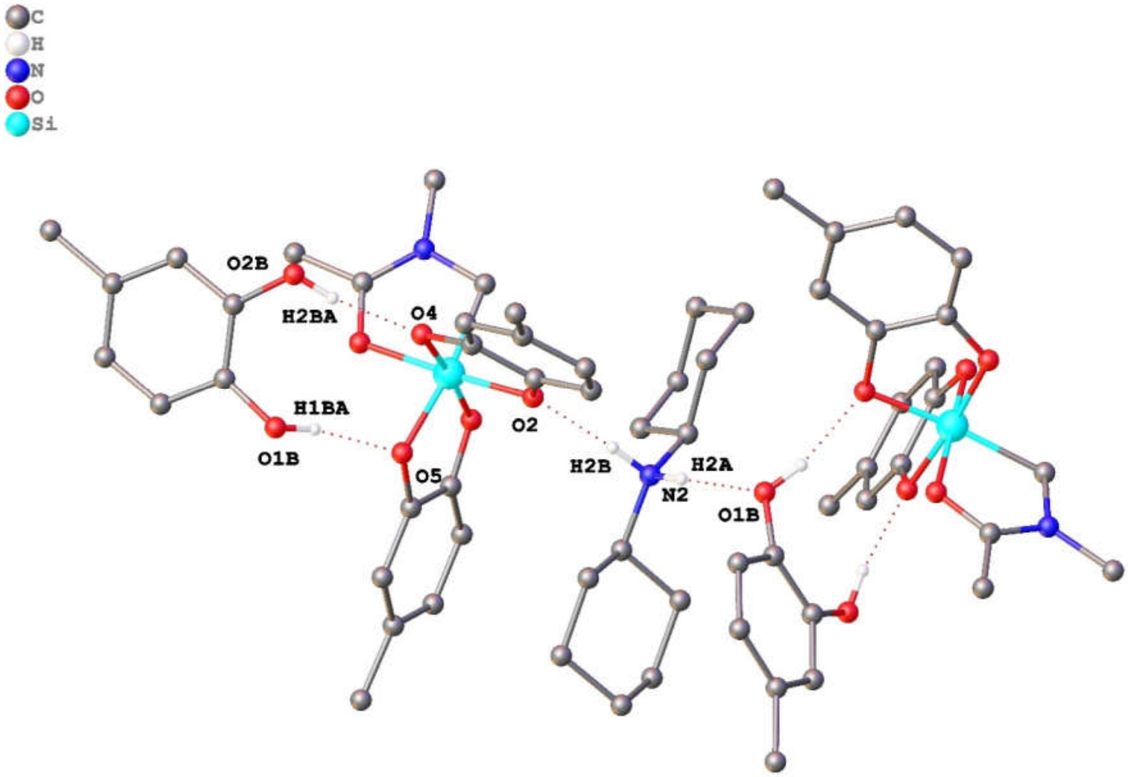

Figure S1. H-bonded chains in crystal structure of **1**.

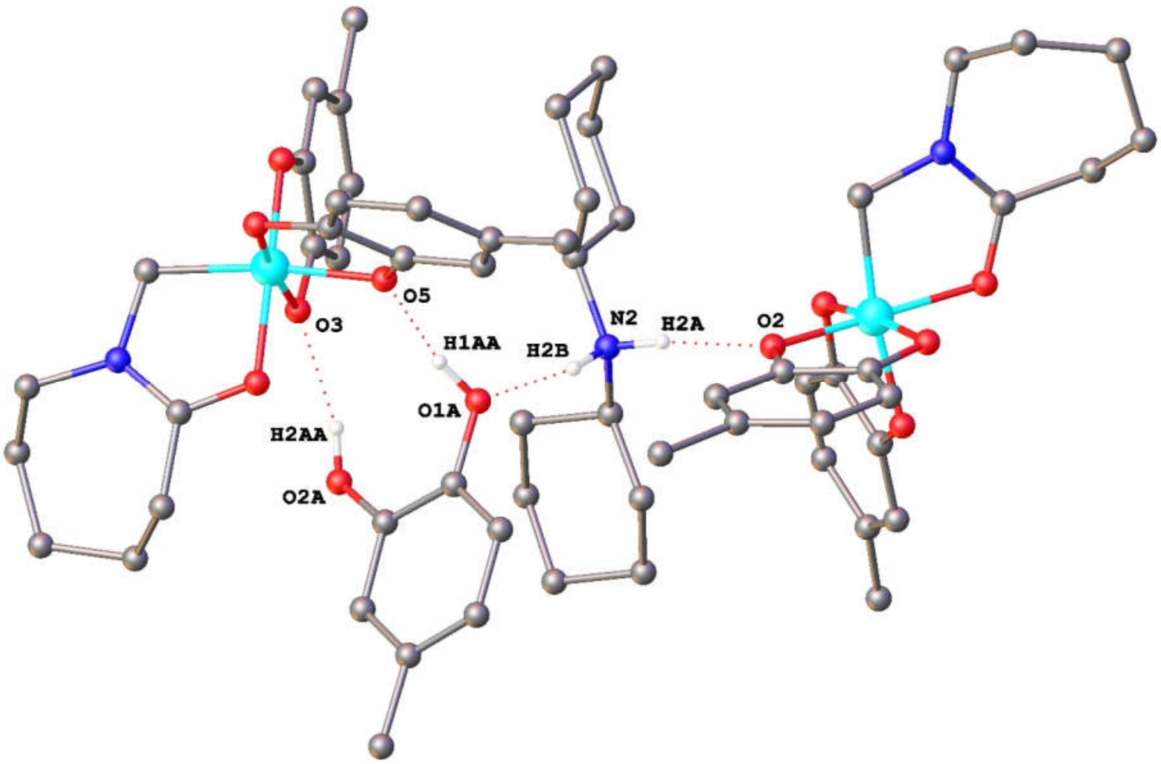

Figure S2. H-bonded chain in crystal structure of **2**.

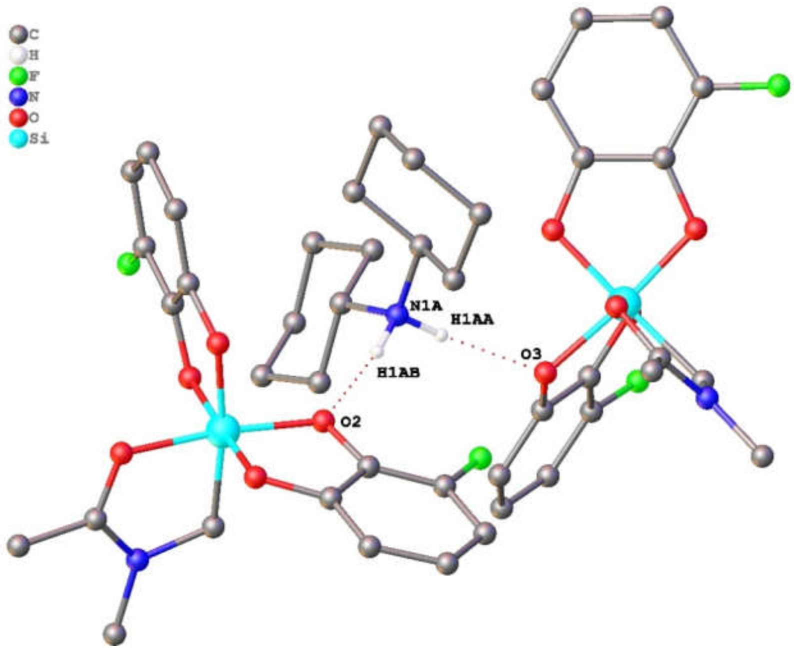

Figure S3. Cation-anion H-bonded chain in crystal structure of **7**.

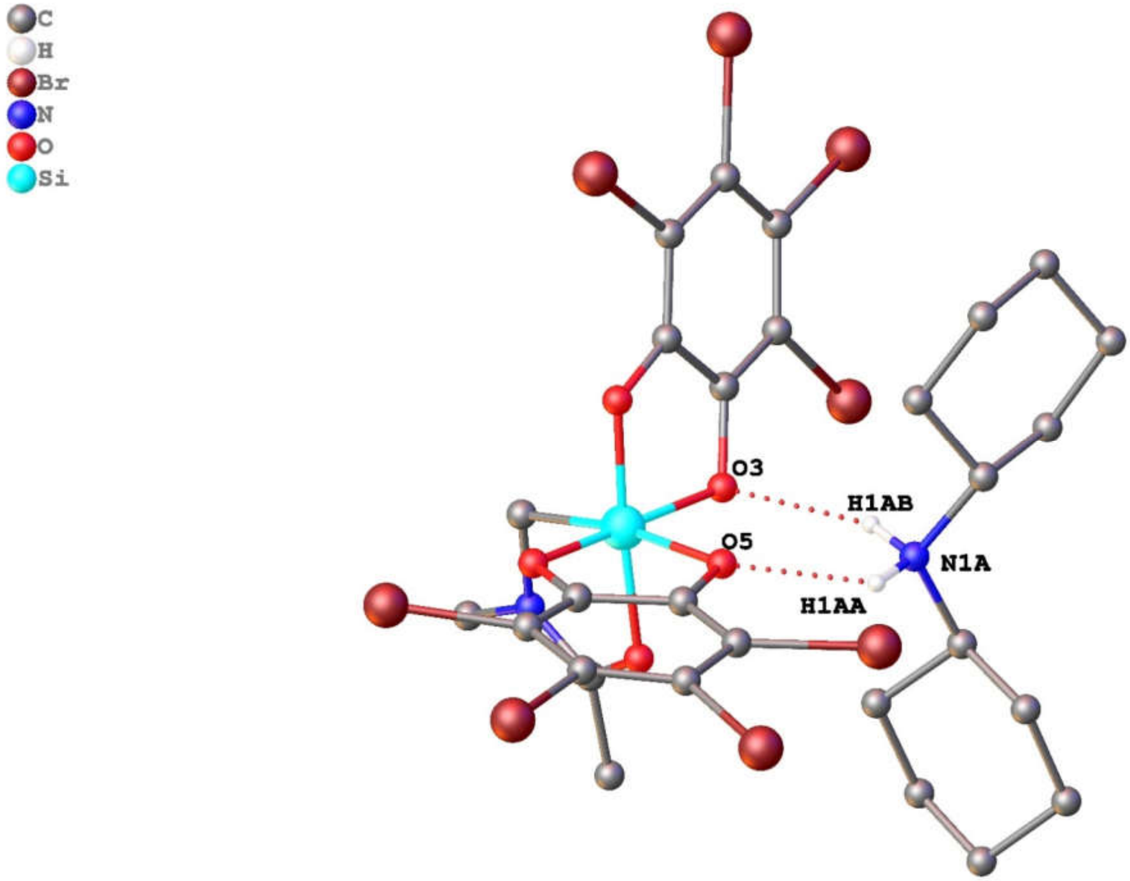

Figure S4. Cation-anion pair in crystal of **4**.

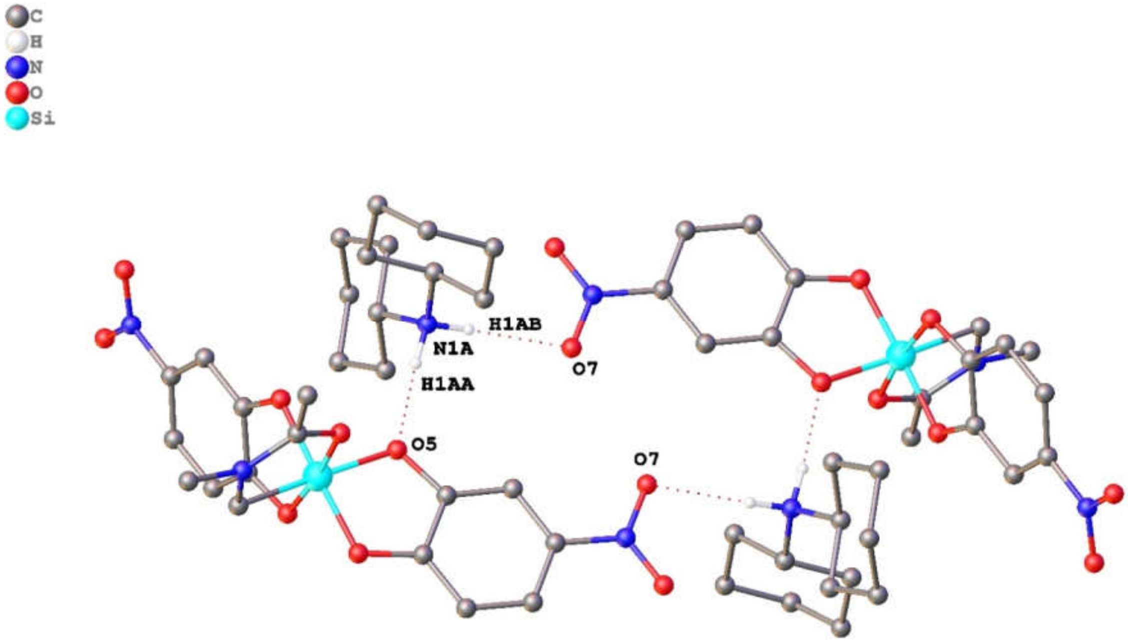

Figure S5. Cation-anion tetramer in crystal of **6**.

In **3a** and **3b**Br…Br and staking interactions between 2,3,4,5-tetrabromocatehol ligands play crucial role in their crystal packing. Indeed, Br…Br and stacking interactions between anions and potassium cations in 6 and **7** construct 2D sieve structure (Figures 6S and 7S). The N[cyc]$_2^+$ cations and solvent molecules are located in inner cells of this sieve network. The structures **3a** and **3b**have almost the same composition, the only difference is the nature of solvate molecules. The first structure contains two solvated acetonitrile molecules while in **3b** one of acetonitrile is replaced by water. This replacement noticeably changes the nature of H-bonds. In **3a** cations and anions form dimers via N-H…O and N-H…Br bonds (Figure 8S). At the same time, in **3b** solvated water molecule serves as spacer between cation and anion forming two O-H…O bonds with oxygen atoms of 2,3,4,5-tetrabromocatehol ligand (Figure 9S). In turn, water molecules cations and anions assembled into infinite chains.

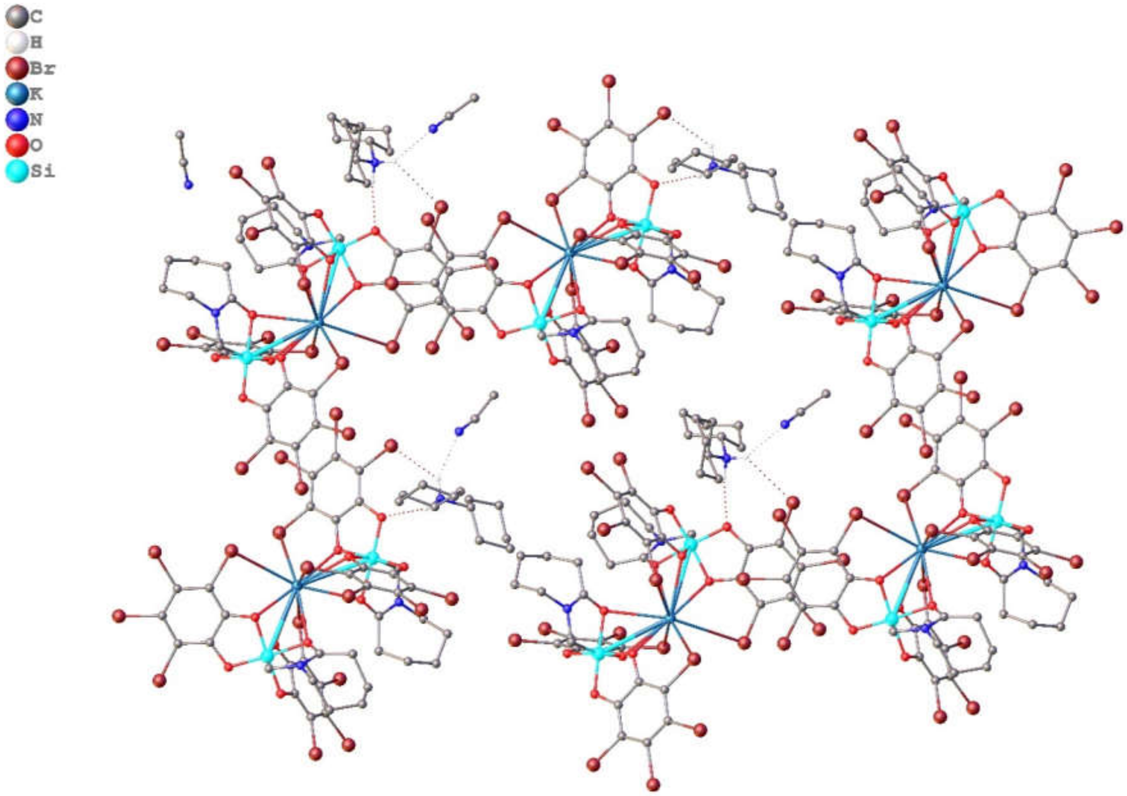

Figure S6. The sieve structure in crystal of **3a**.

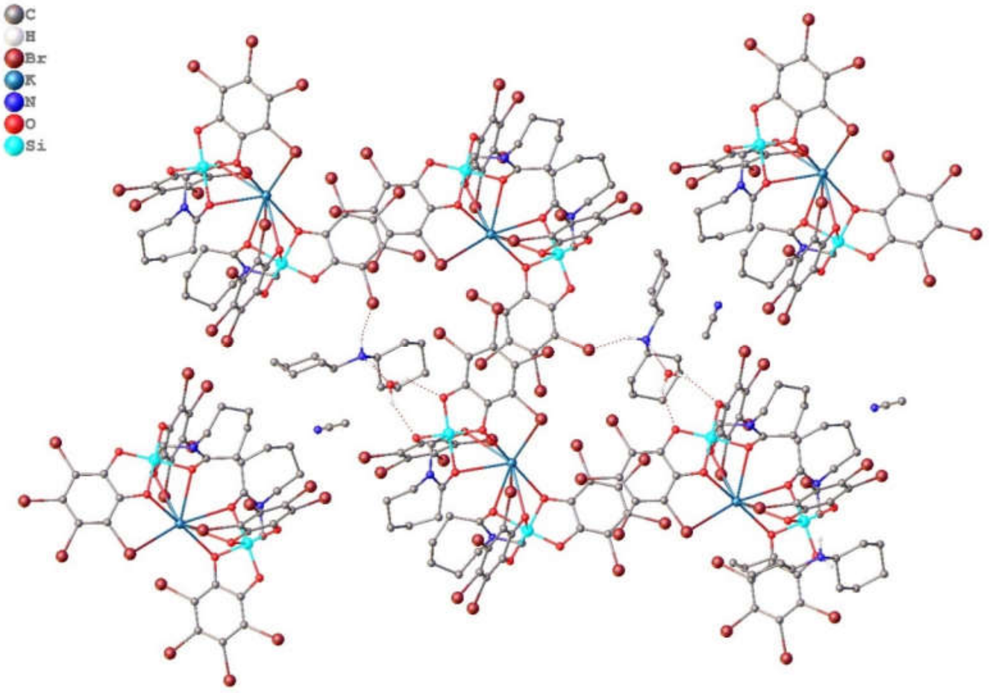

Figure S7. The sieve structure in crystal of **3b**.

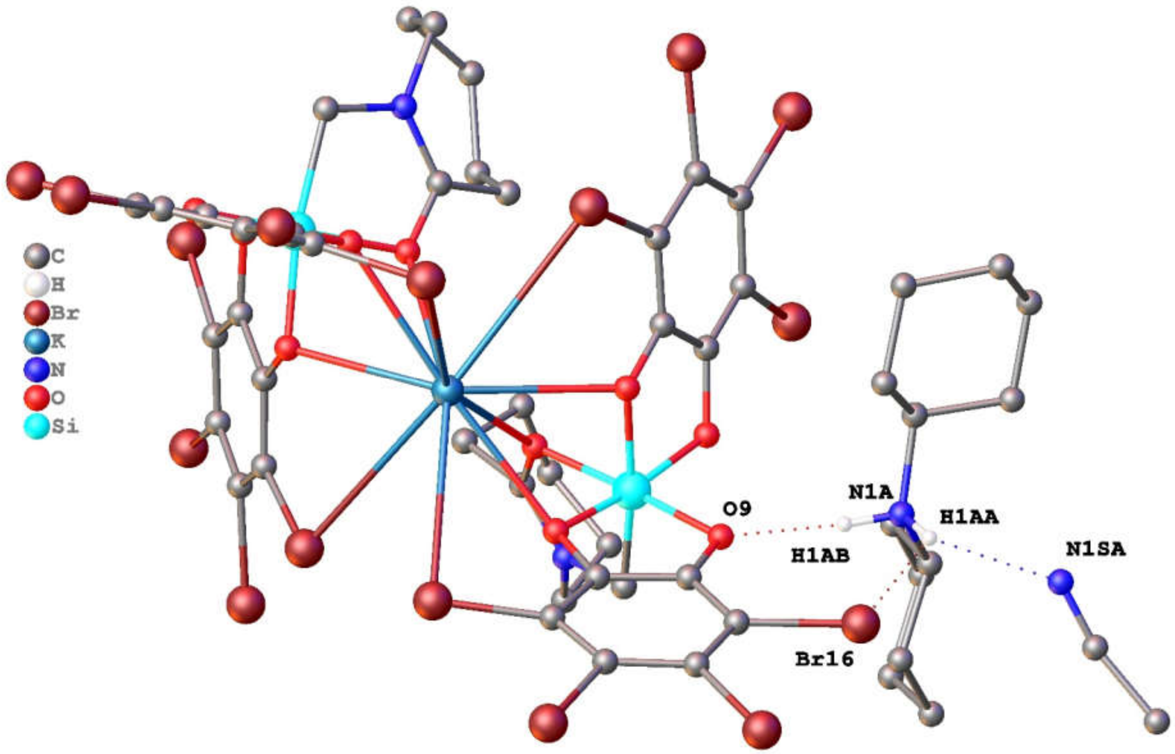

Figure S8. H-bonded chain in crystal of **3a**.

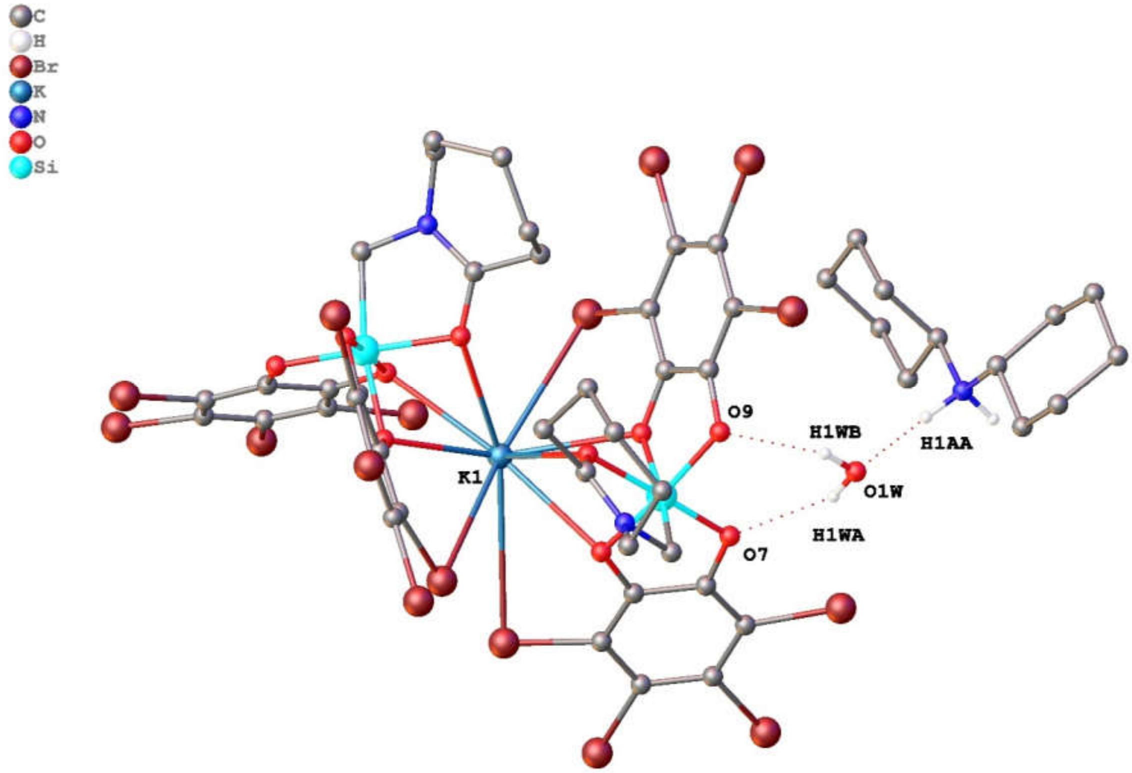

Figure S9. H-bonded chain in crystal of **3b**.

Compound **5** is crystallized with two water molecules. Cations, anions and water molecules in crystal packing of **5** form chains via O-H…O and N-H…O bonds. In turn, these dimers are assembled into 3D network via N-H…O bond between one of water molecule and cyanogroup of substituted catecholate ligand.

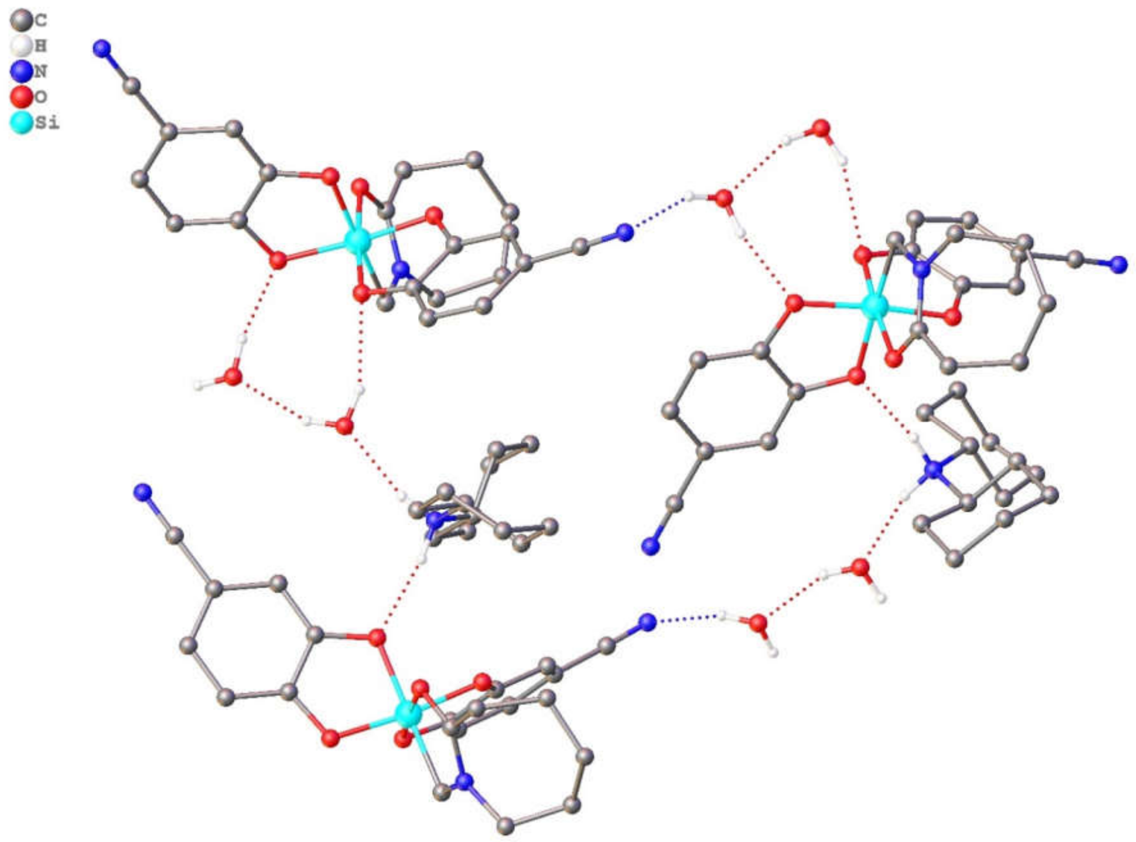

Figure S10. H-bonded cycle formed by cations and anion with inclusion of solvated water molecules in crystal of **5**.

CV diagrams for compounds 1–7

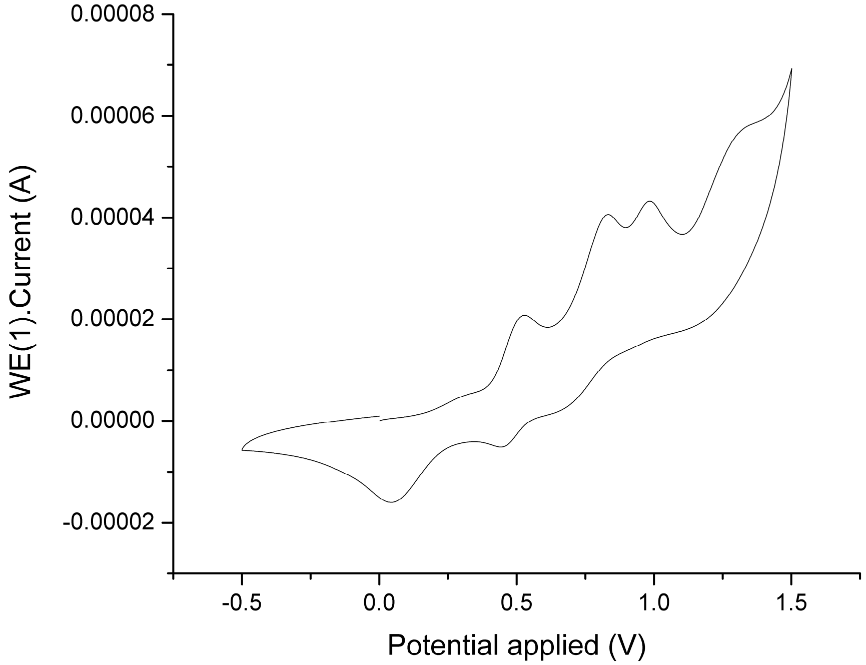

Figure S11. CV diagram of **2.**

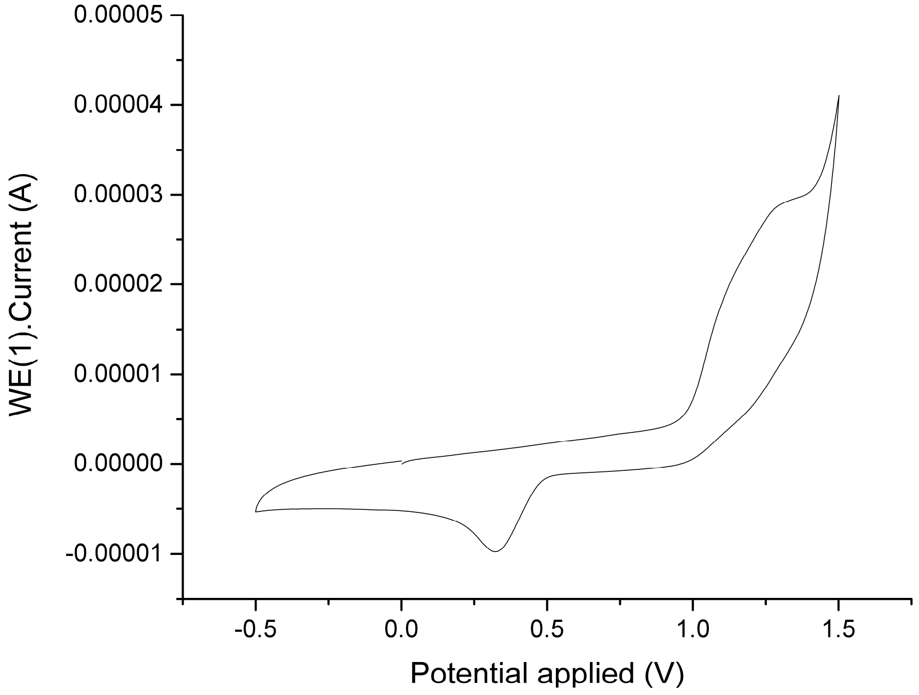

Figure S12. CV plot of **3a.**

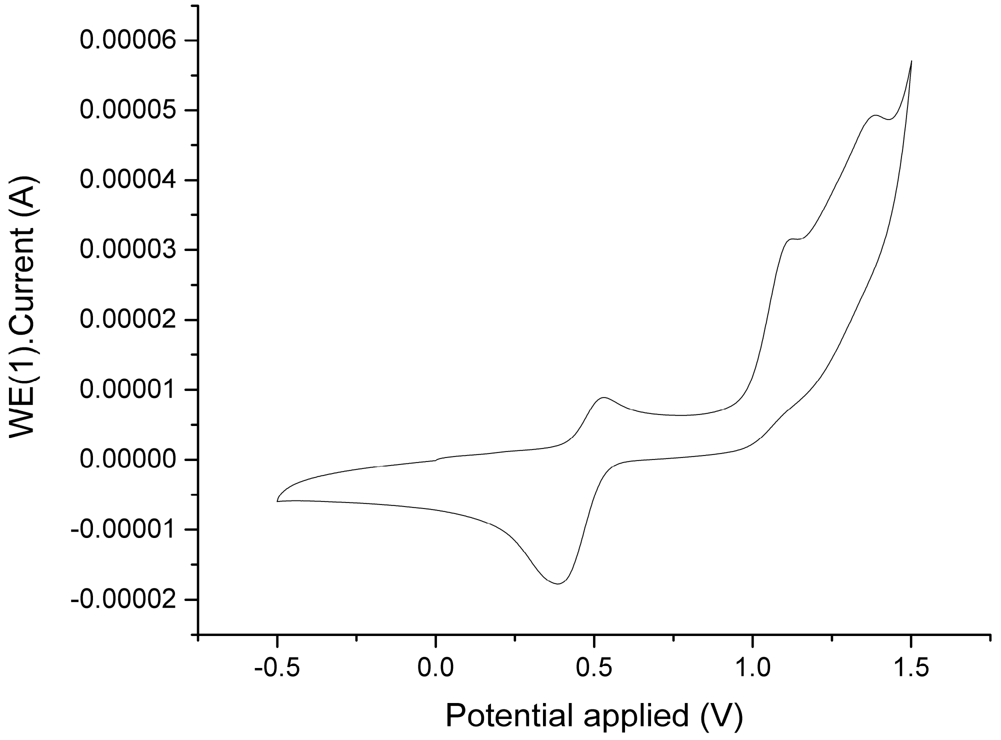

Figure S13. CV plot of **4.**

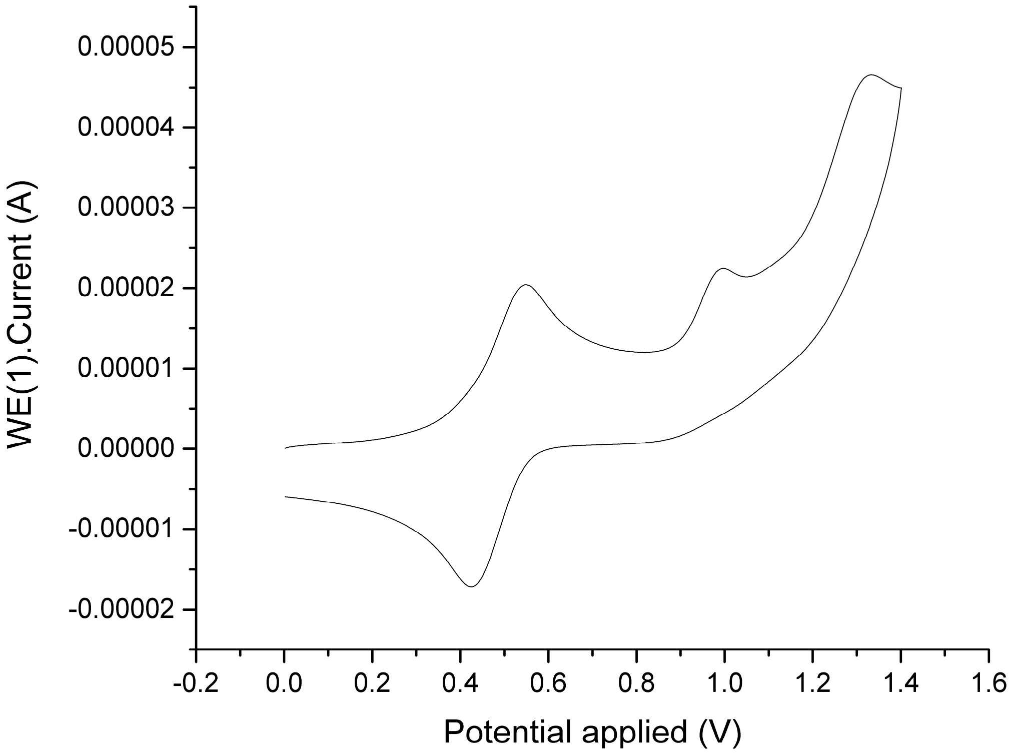

Figure S14. CV plot of **5.**

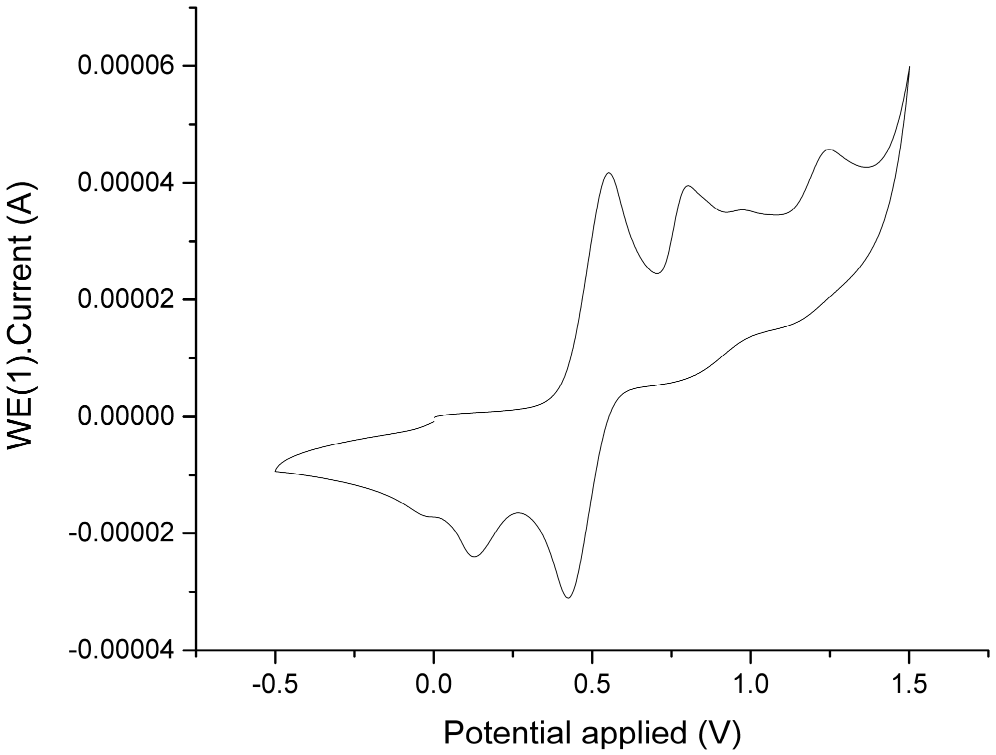

Figure S15. CV plot of **7**.

1. H, ^{13}C, ^{29}Si NMR and HRMS spectra

^{1}H NMR spectrum of compound **1.**

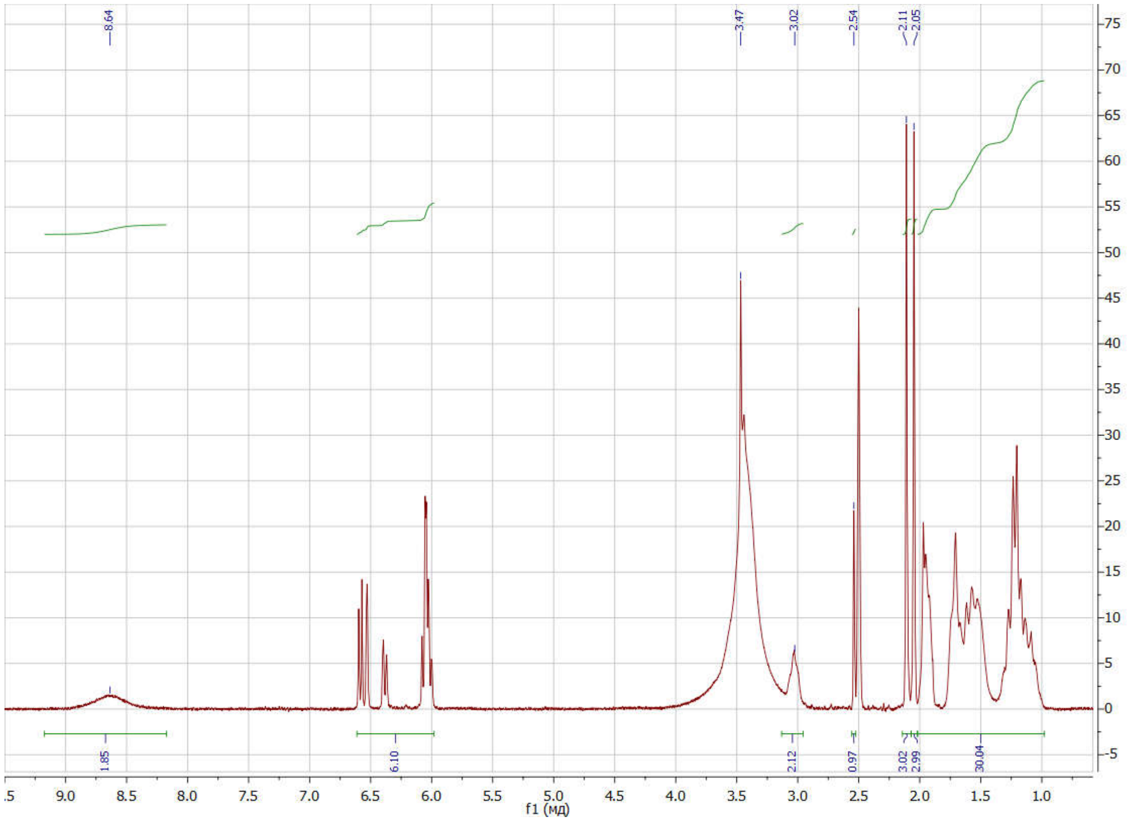

^{13}C NMR spectrum of compound **1.**

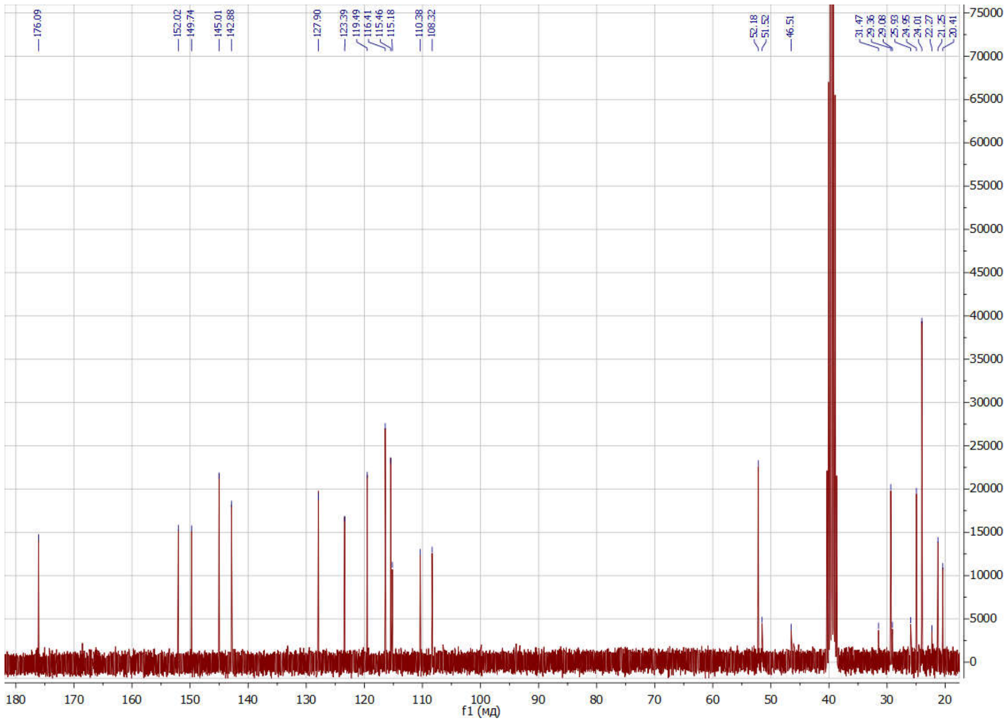

^{29}Si NMR spectrum of compound **1.**

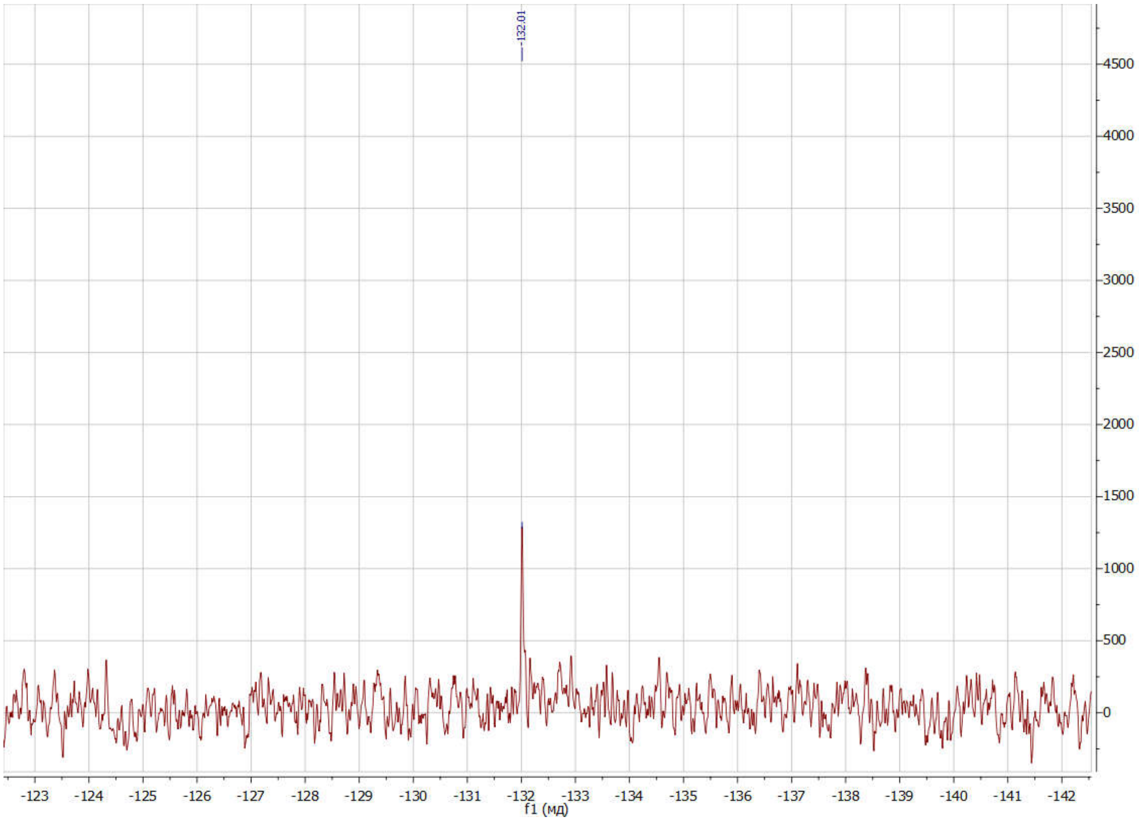

HRMS spectrum of compound **1.**

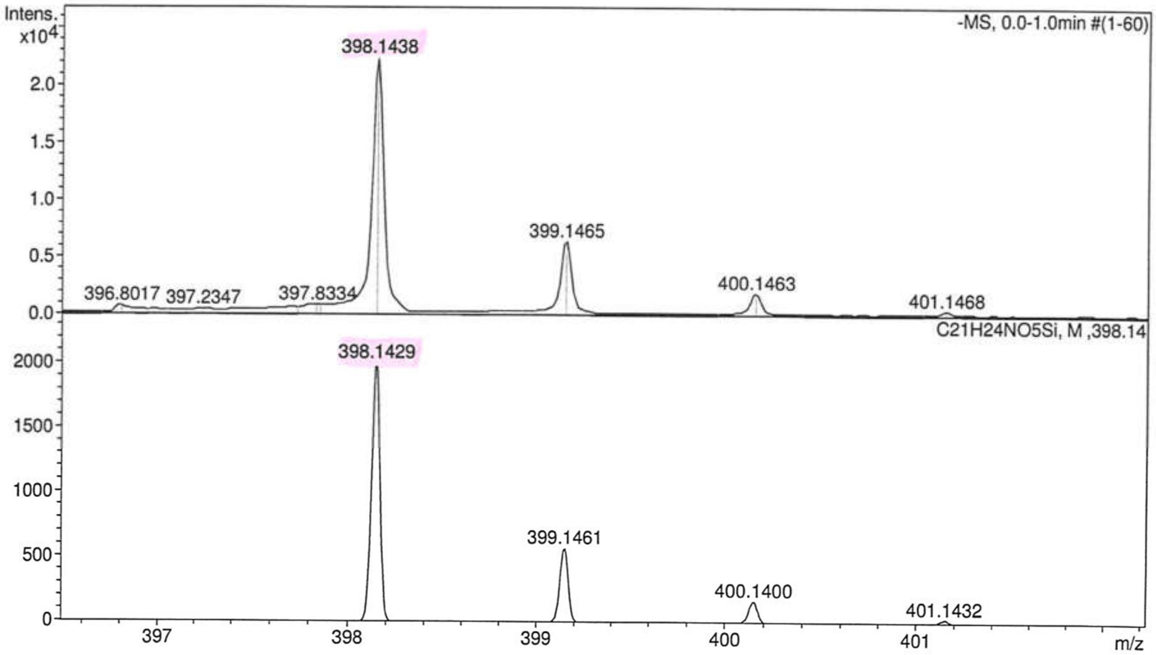

^{1}H NMR spectrum of compound **2.**

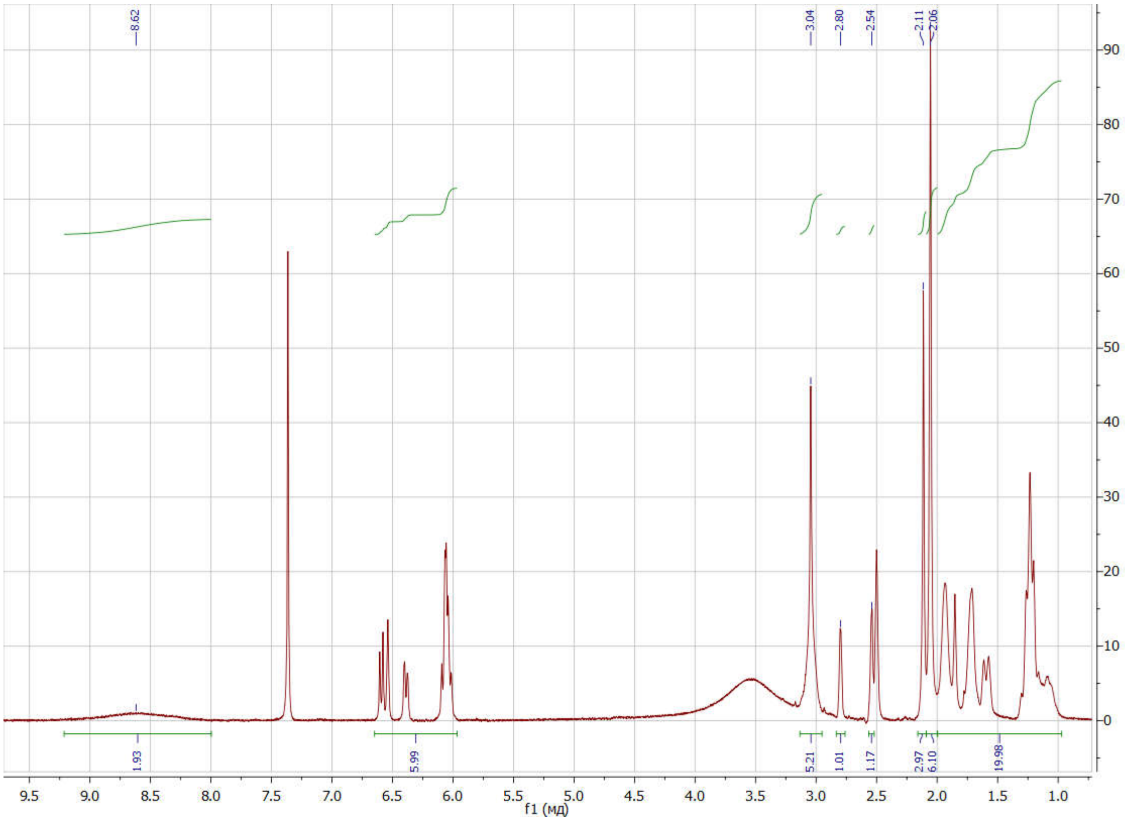

^{13}C NMR spectrum of compound **2.**

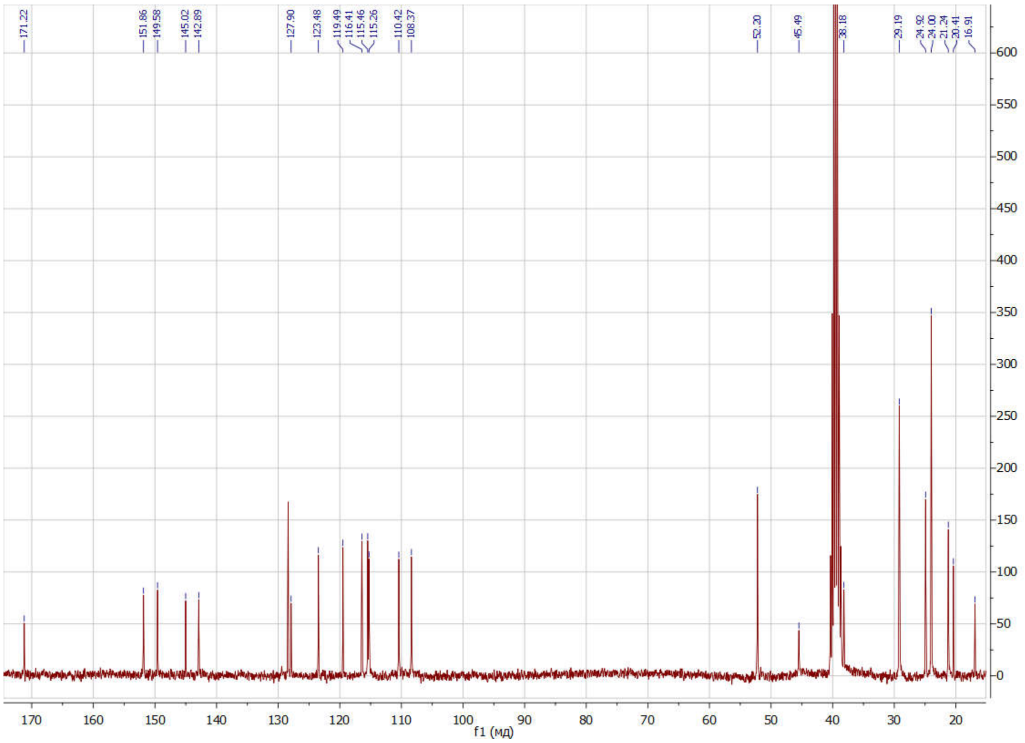

^{29}Si NMR spectrum of compound **2.**

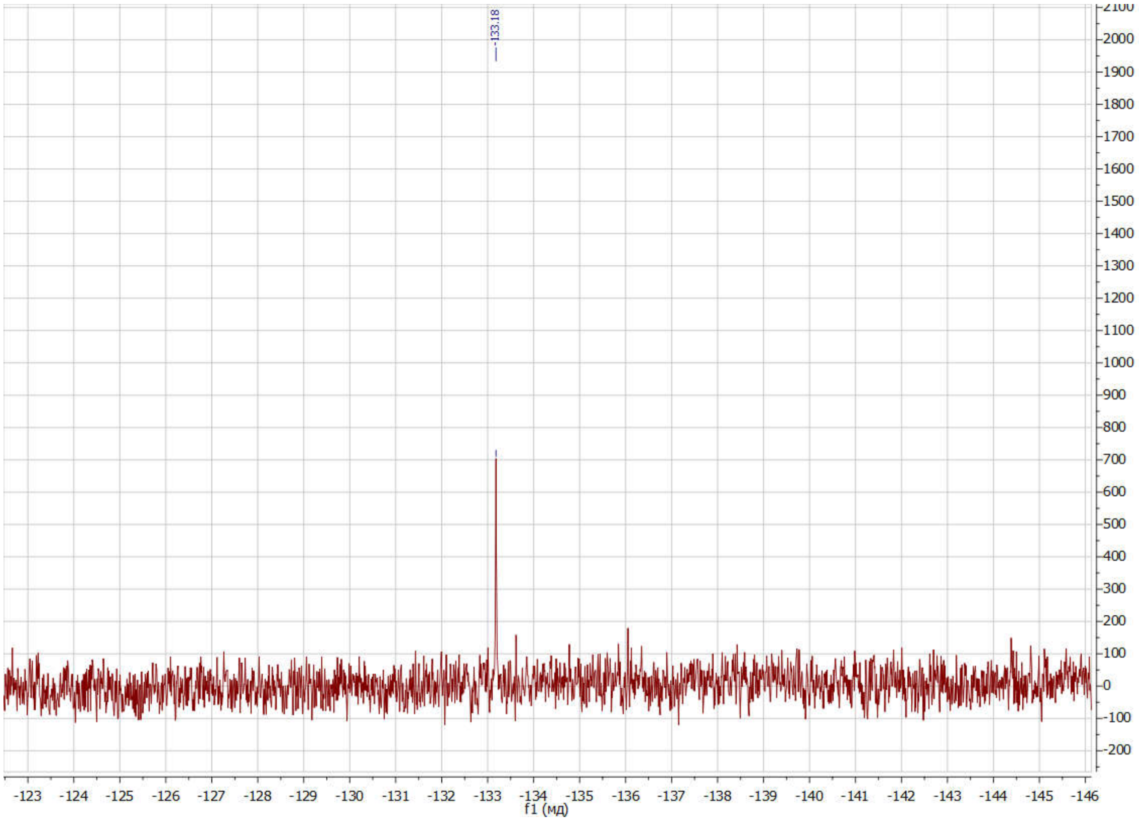

HRMS spectrum of compound **2.**

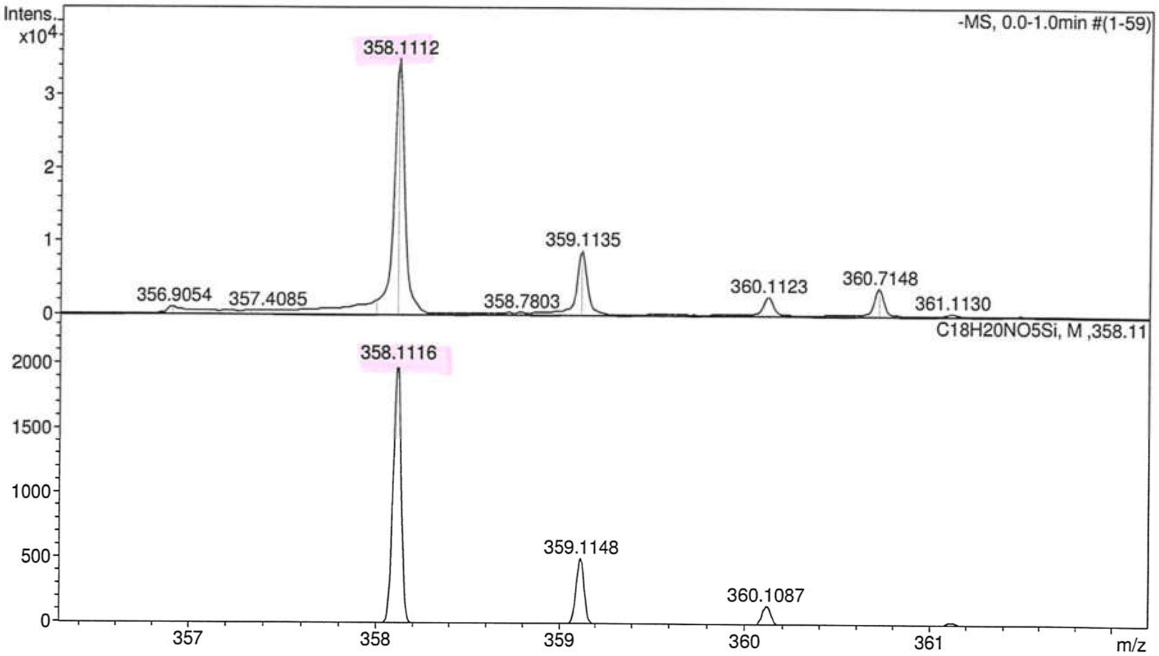

^{1}H NMR spectrum of compound **3.**

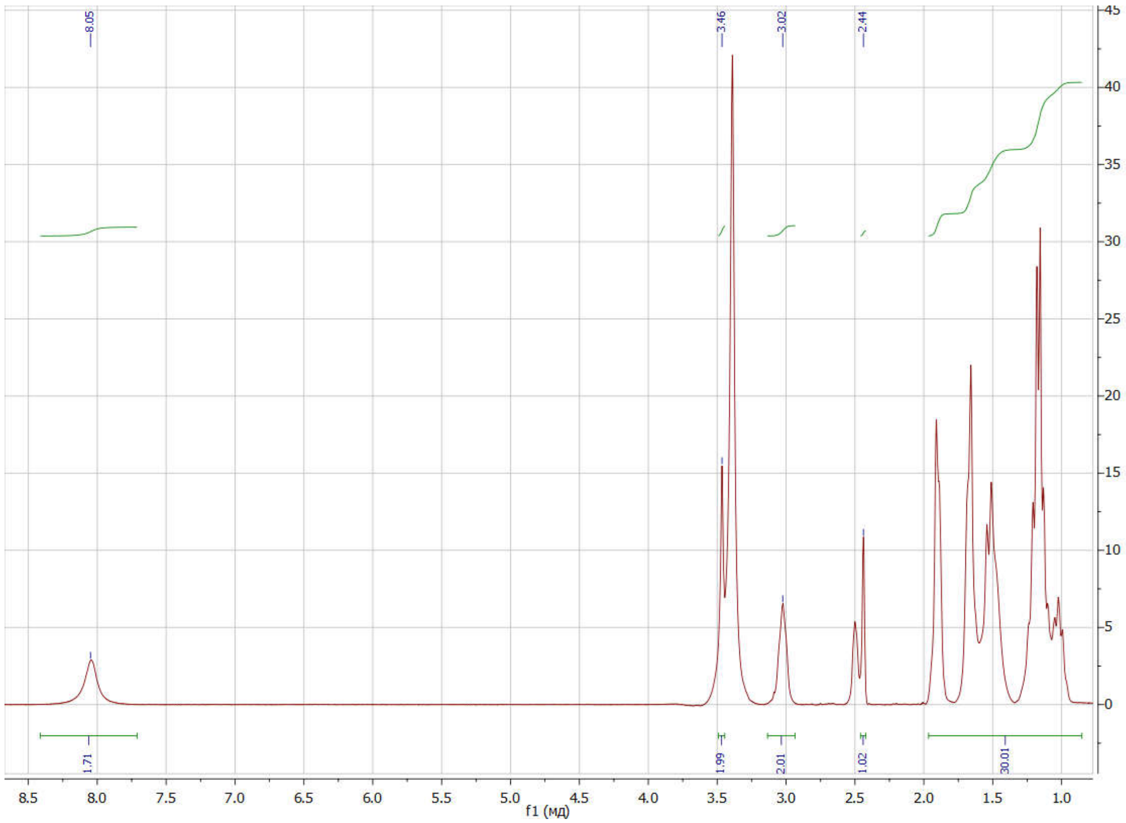

^{13}C NMR spectrum of compound **3.**

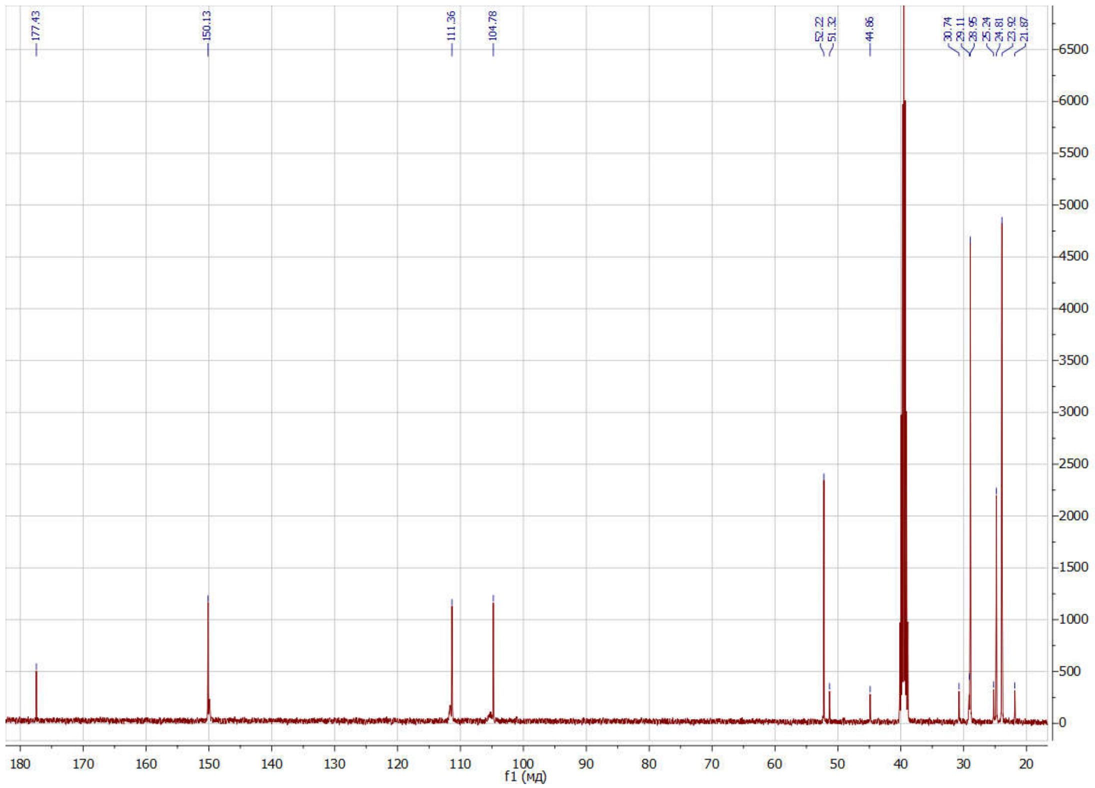

^{29}Si NMR spectrum of compound **3.**

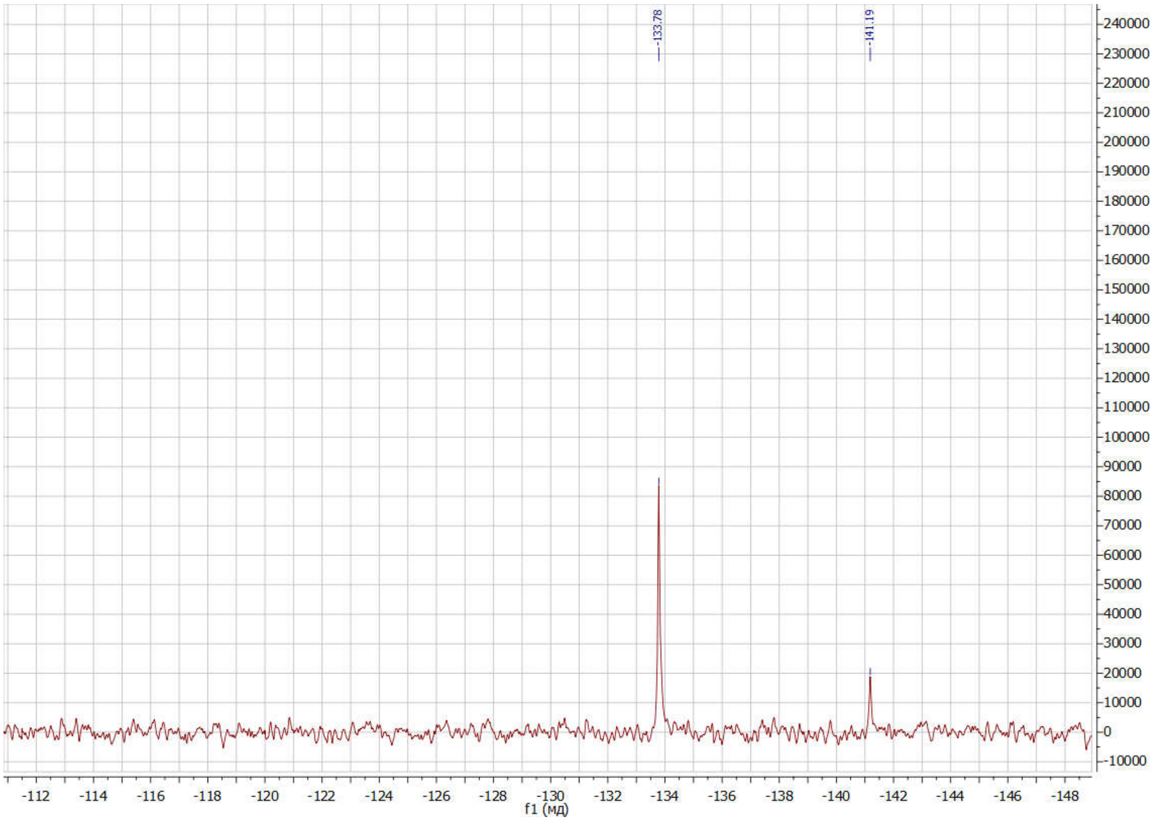

HRMS spectrum of compound **3.**

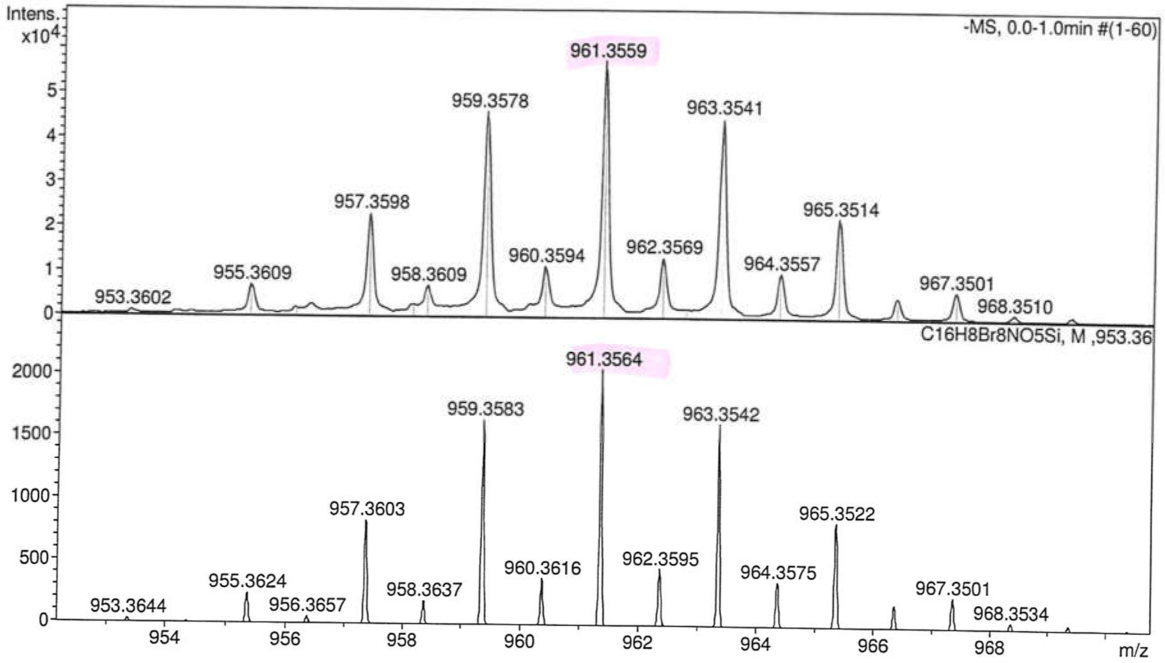

^{1}H NMR spectrum of compound **4.**

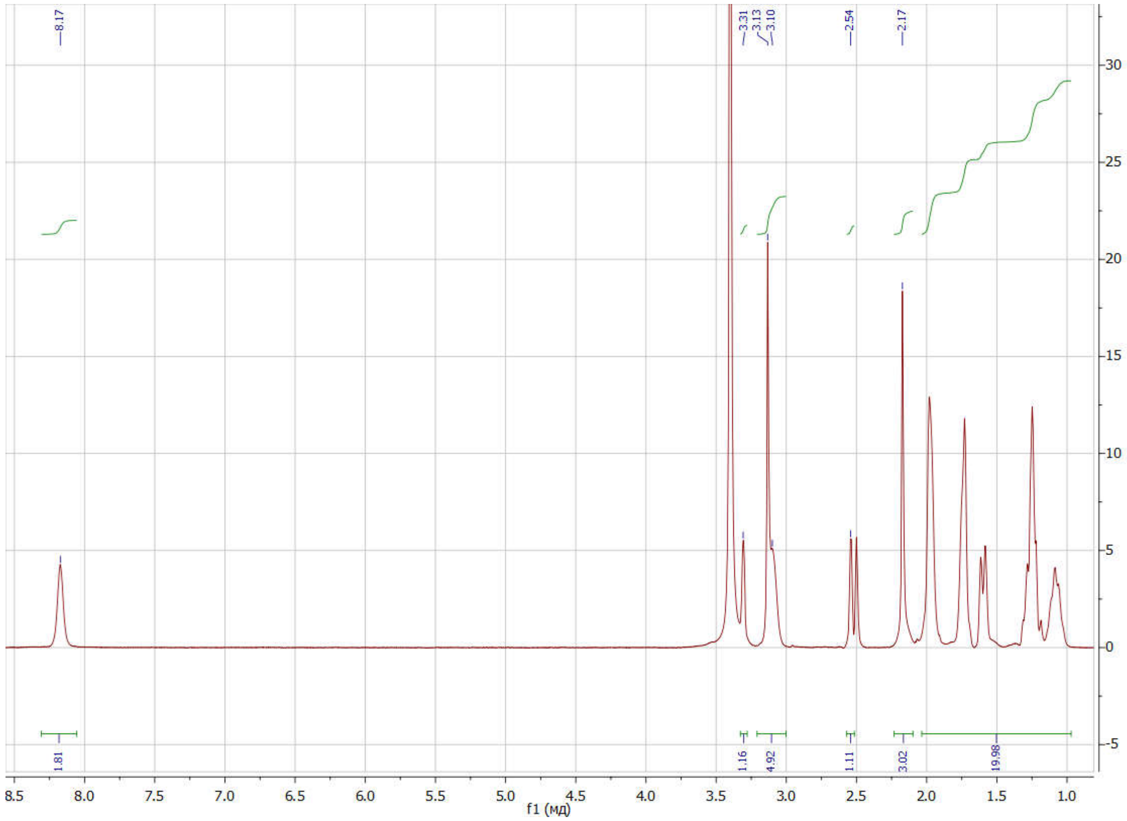

^{13}C NMR spectrum of compound **4.**

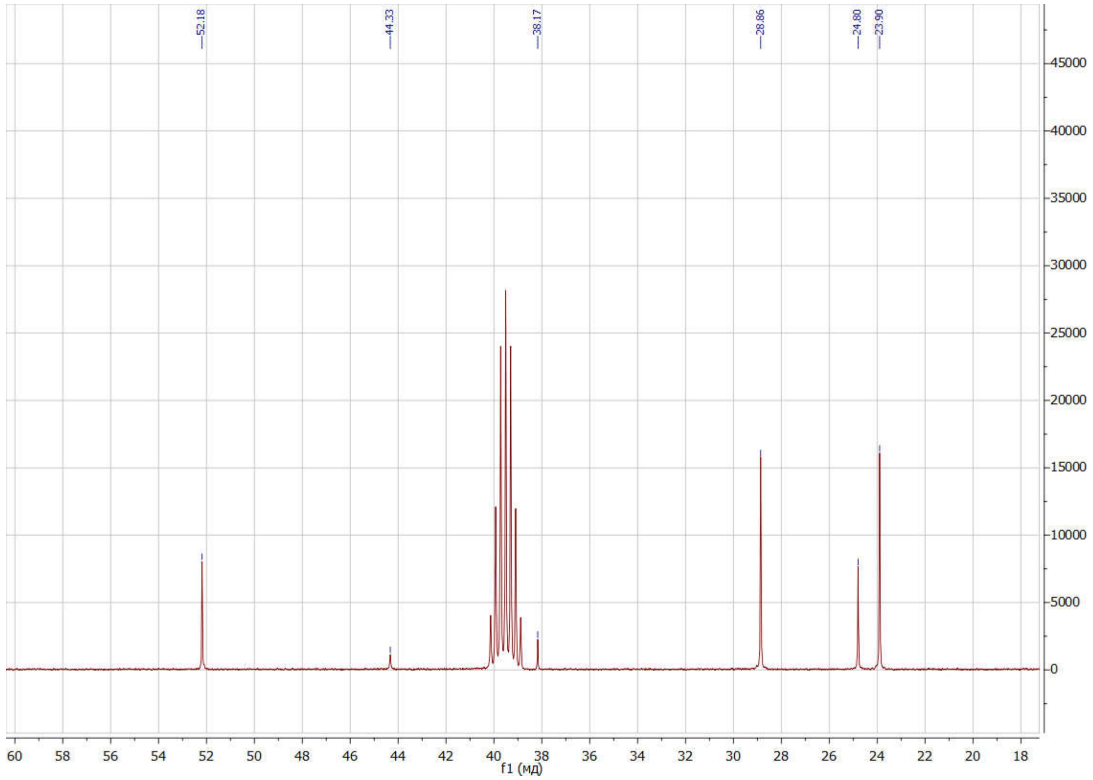

^{29}Si NMR spectrum of compound **4.**

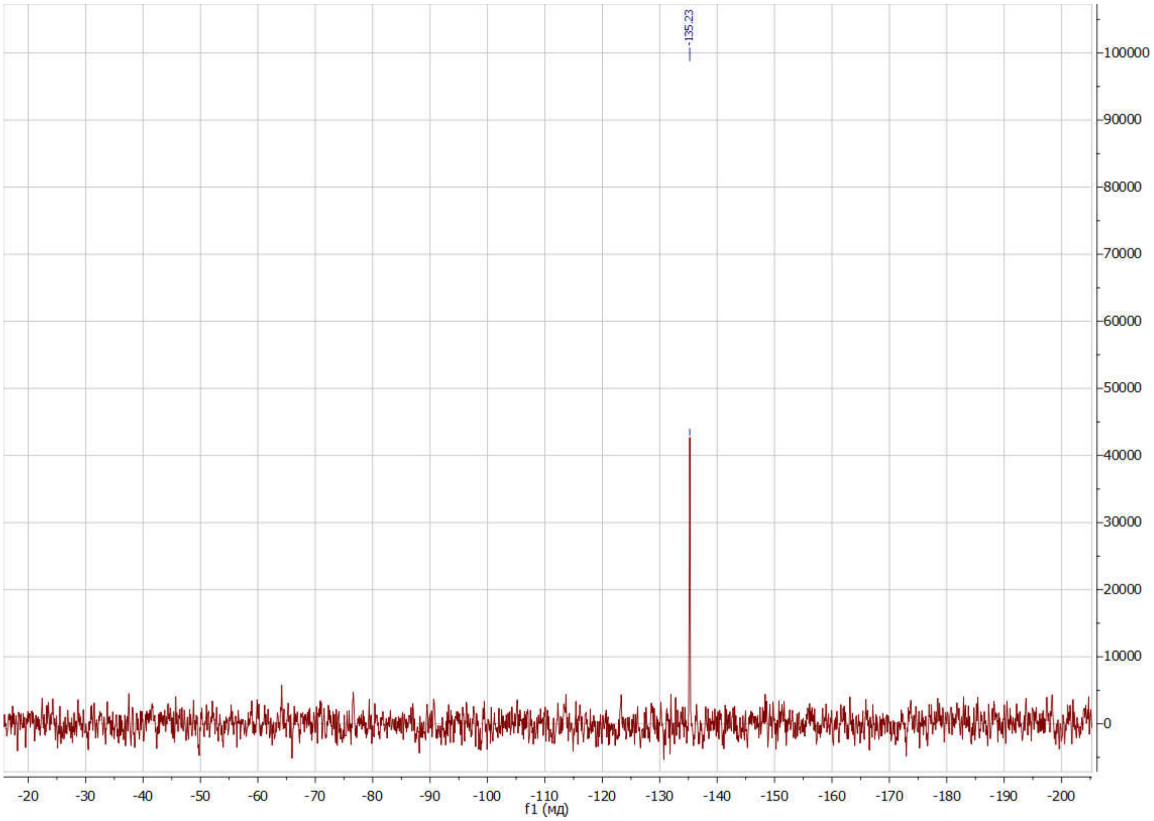

HRMS spectra of compound **4.**

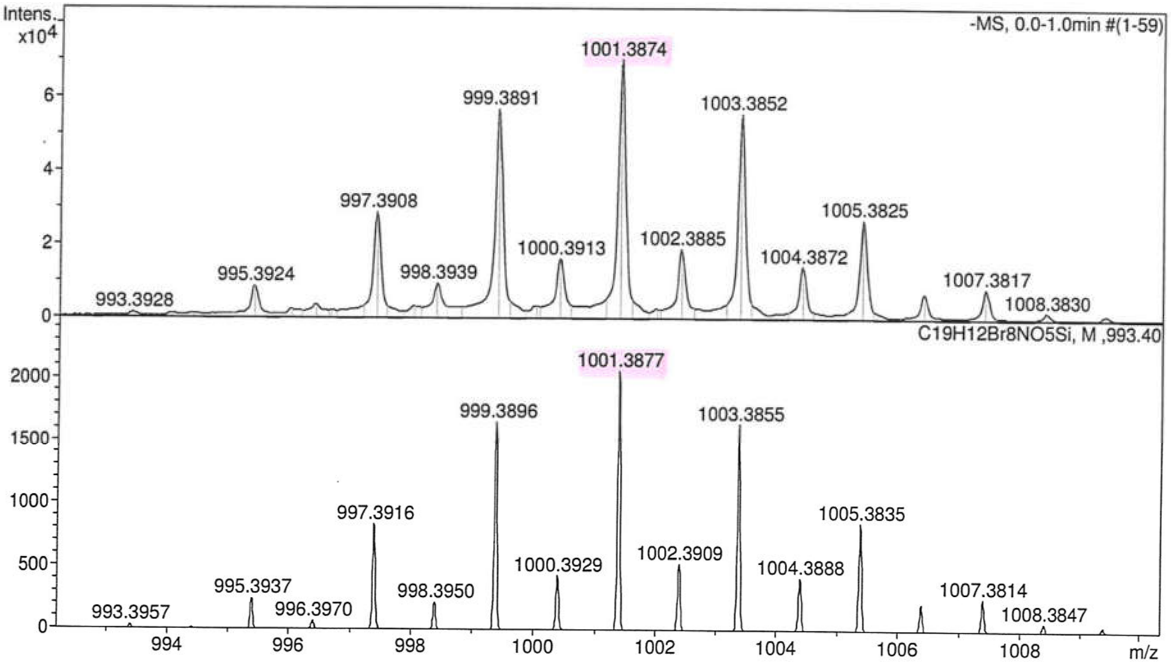

^{1}H NMR spectrum of compound **5.**

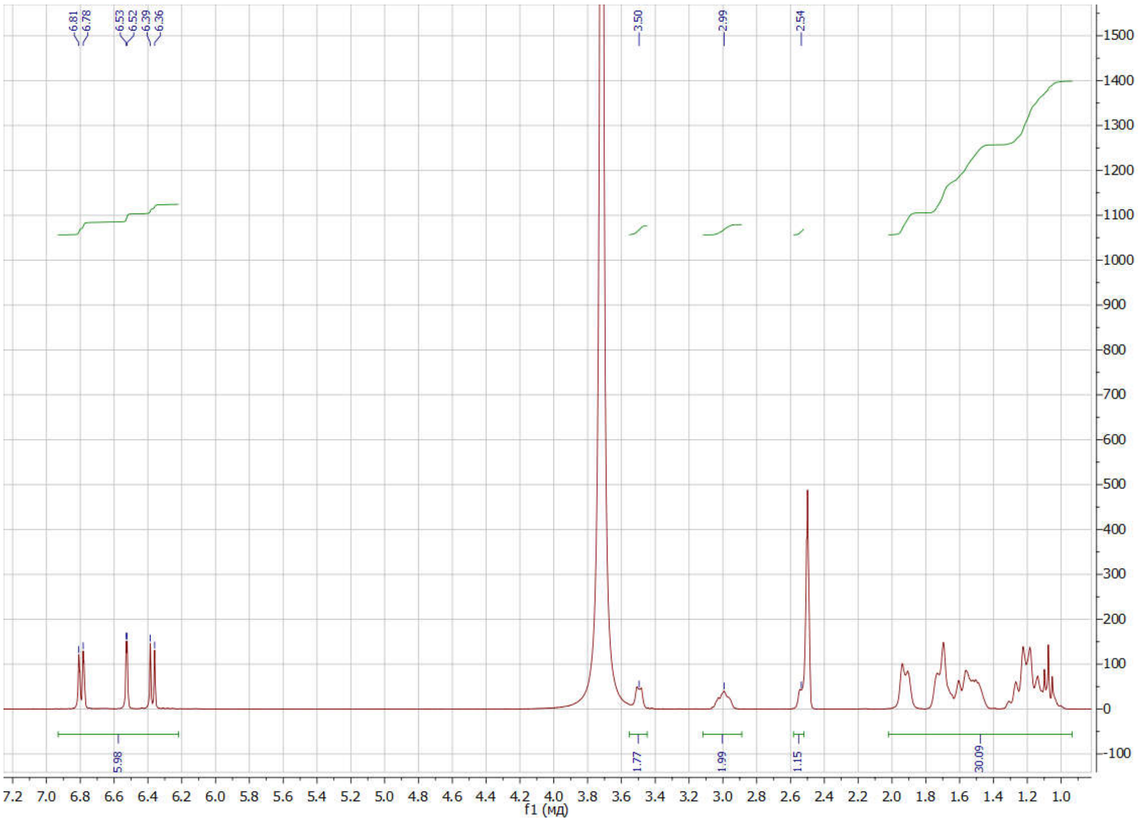

^{13}C NMR spectrum of compound **5.**

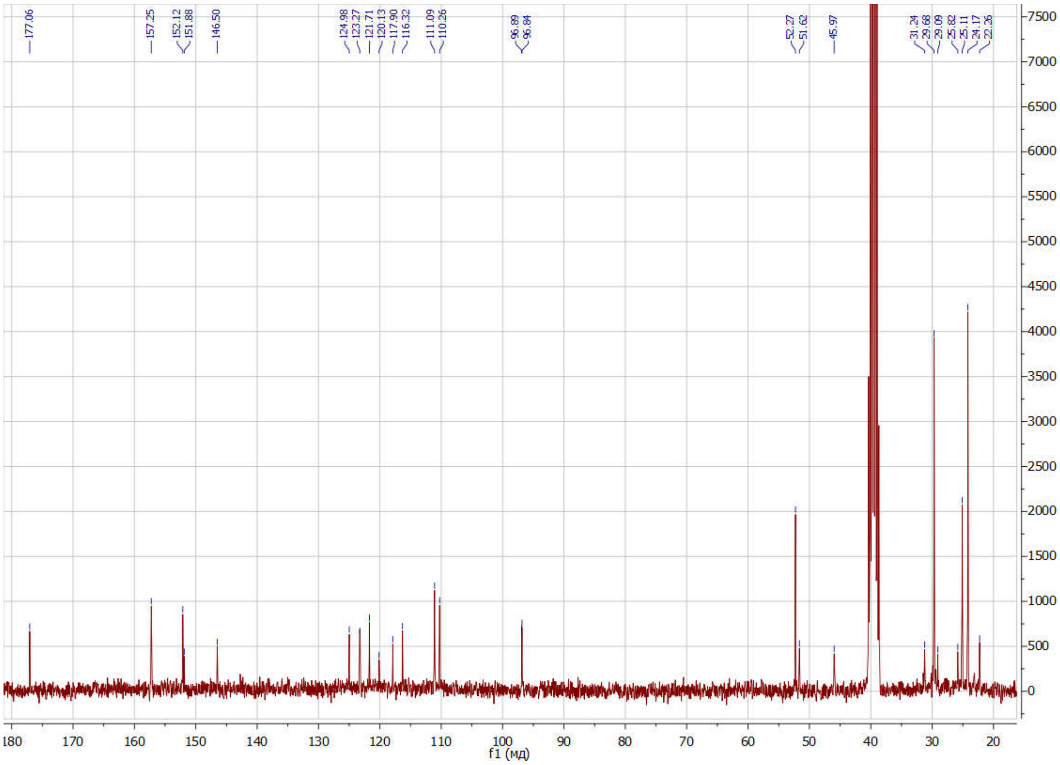

^{29}Si NMR spectra of compound **5.**

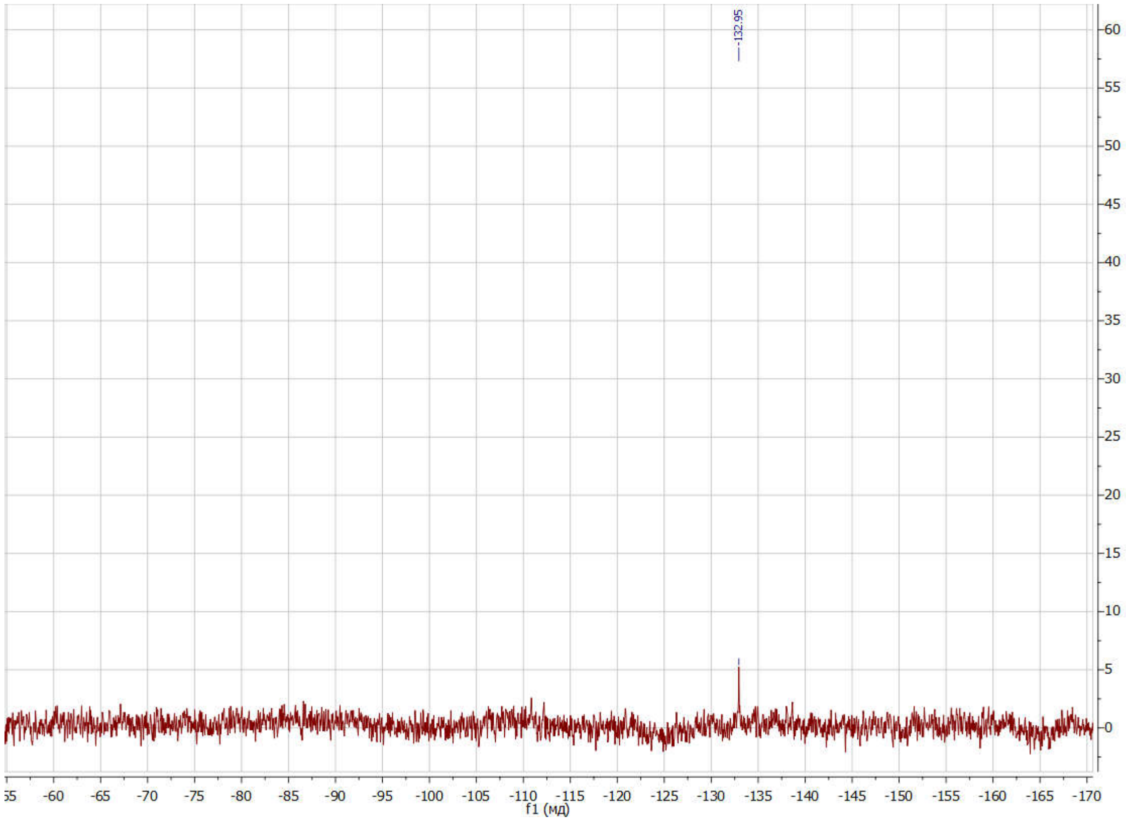

HRMS spectrum of compound **5.**

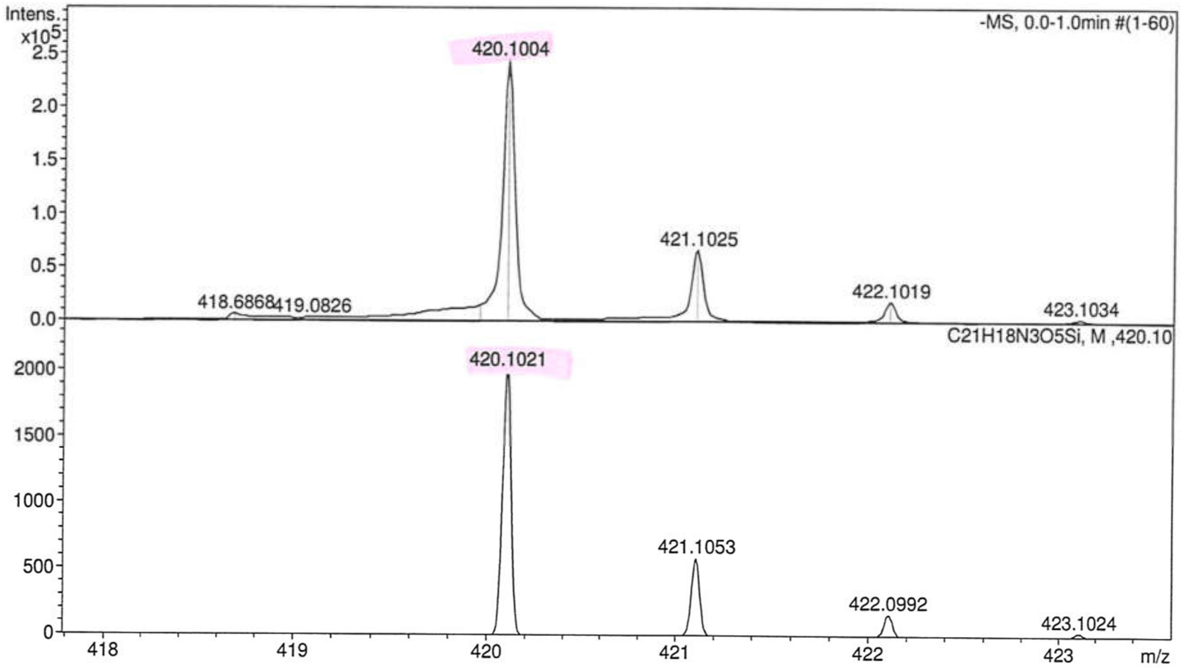

¹H NMR spectrum of compound **6.**

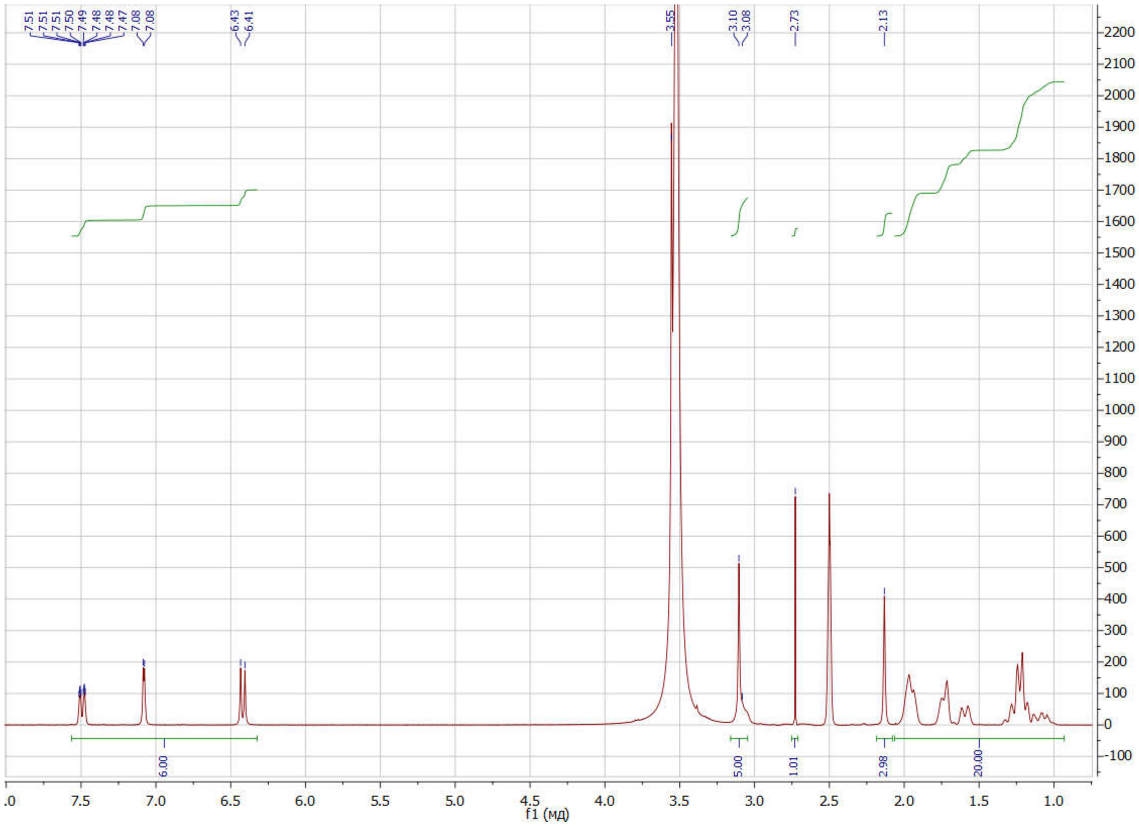

¹³C NMR spectrum of compound **6.**

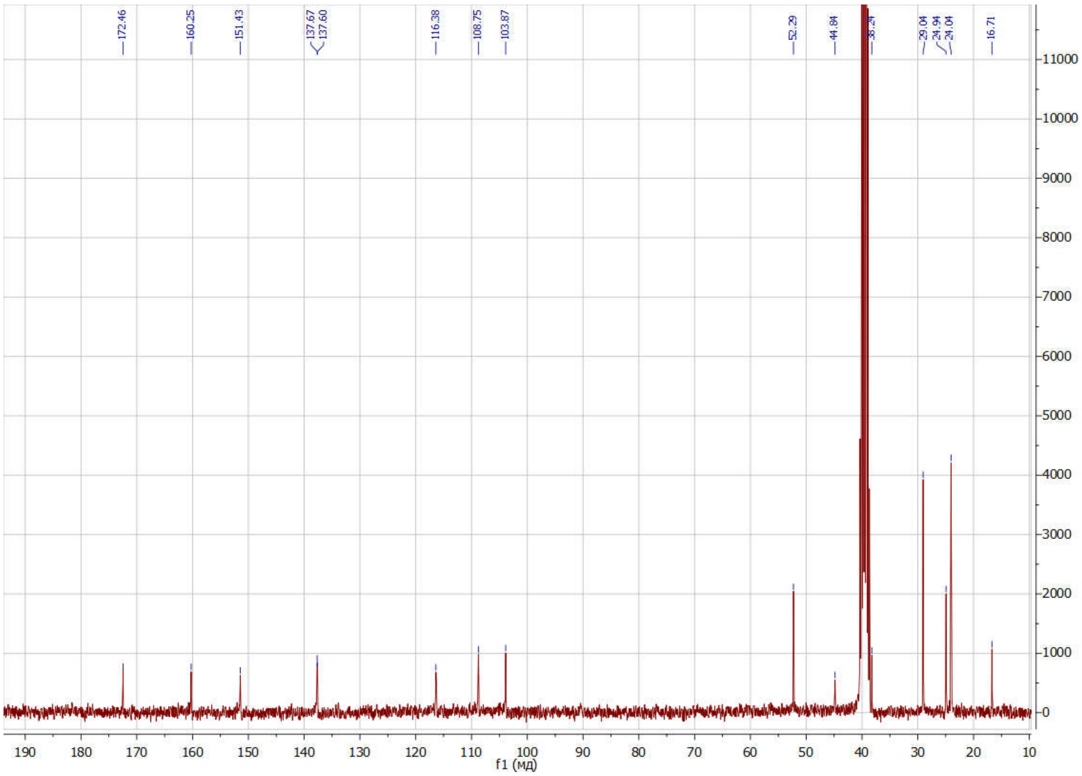

^{29}Si NMR spectra of compound **6.**

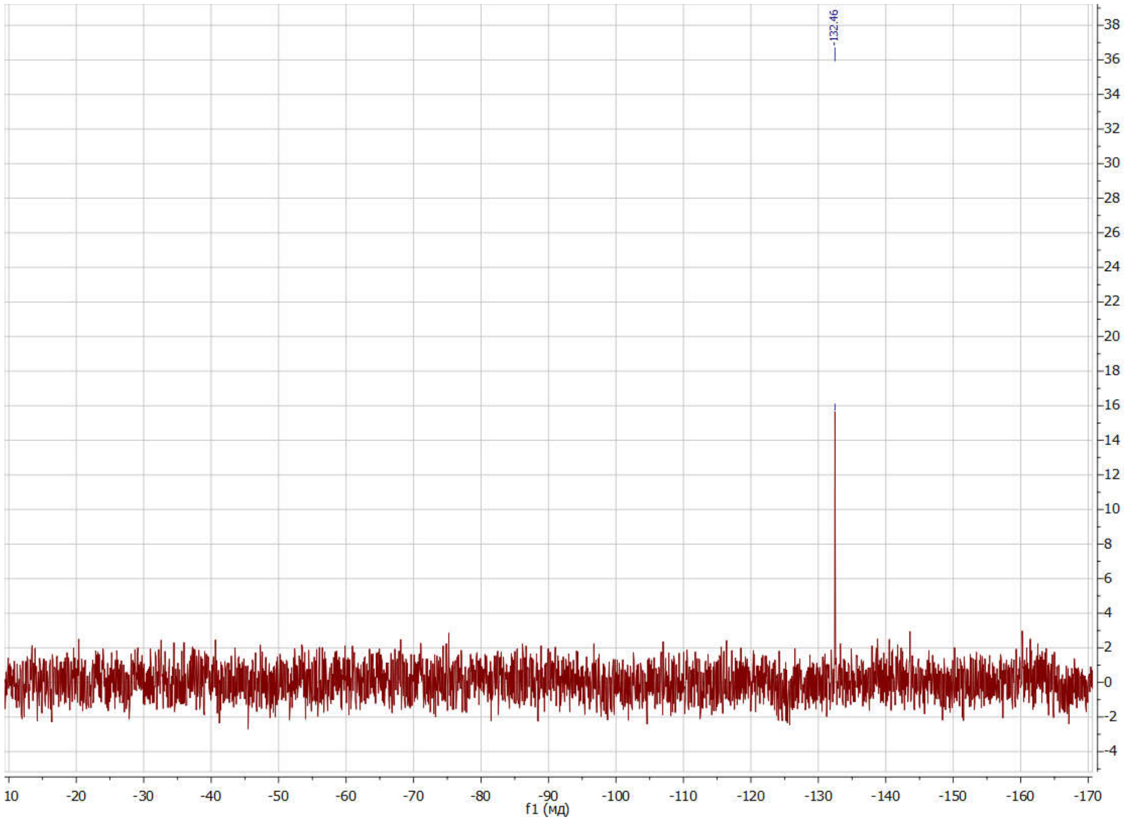

HRMS spectrum of compound **6.**

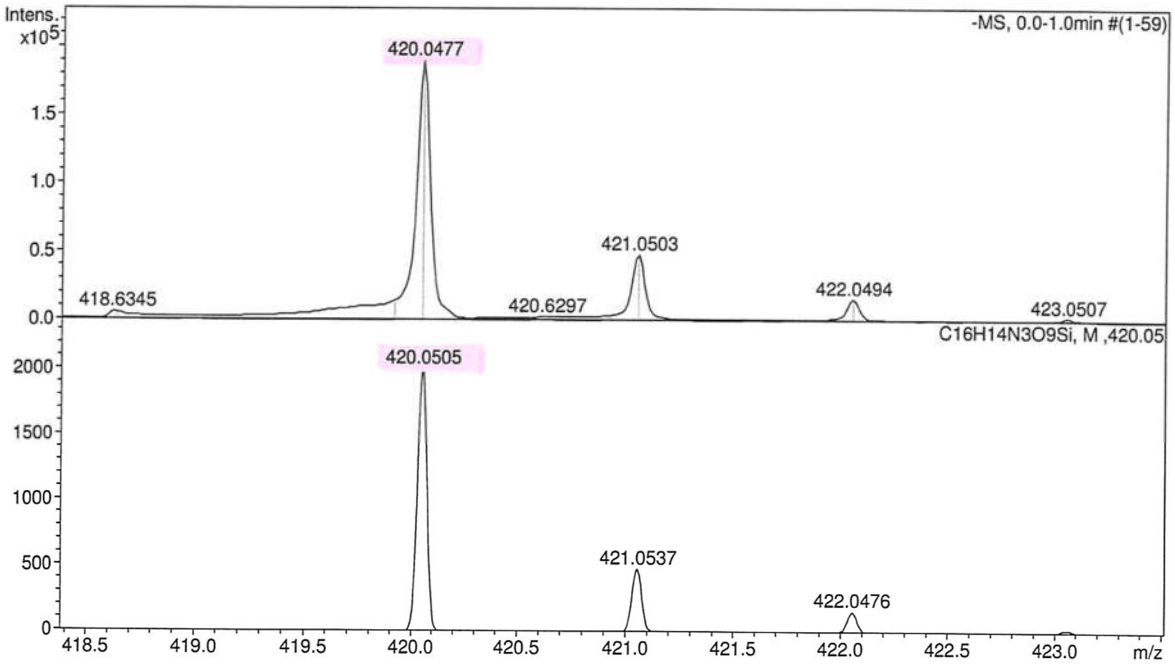

^{1}H NMR spectrum of compound **7.**

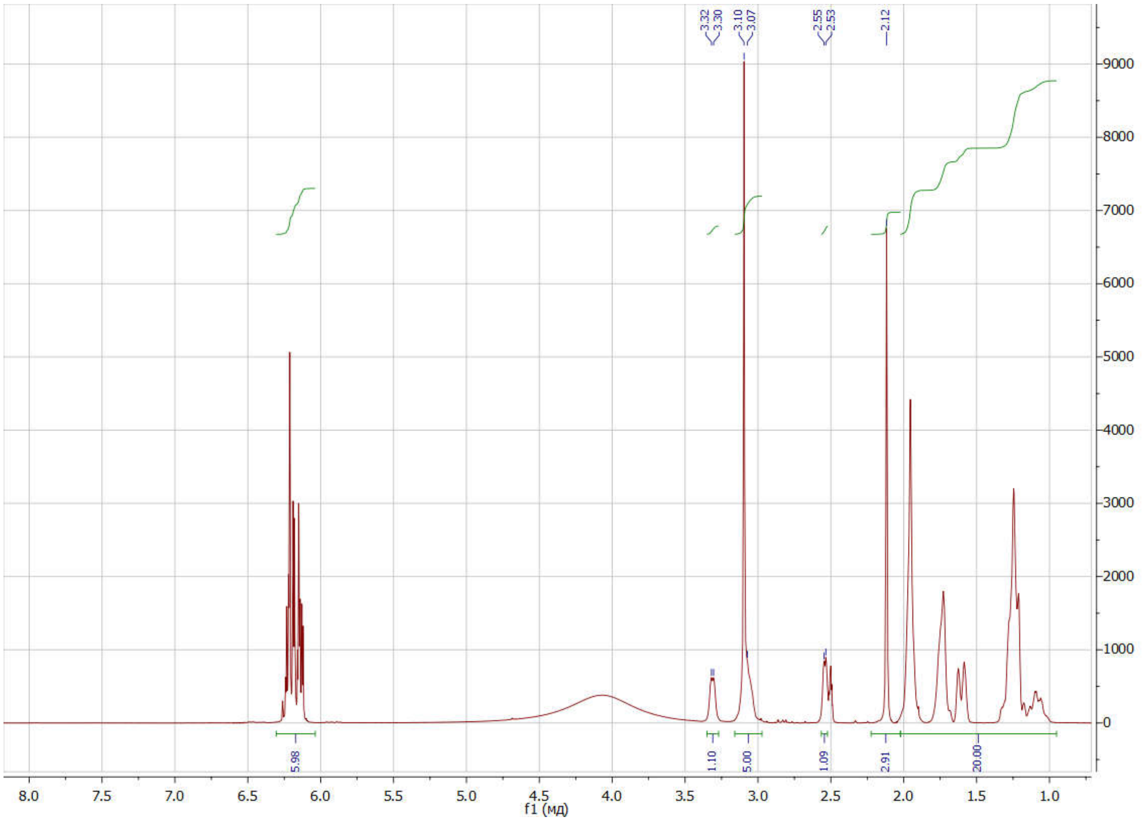

^{13}C NMR spectrum of compound **7.**

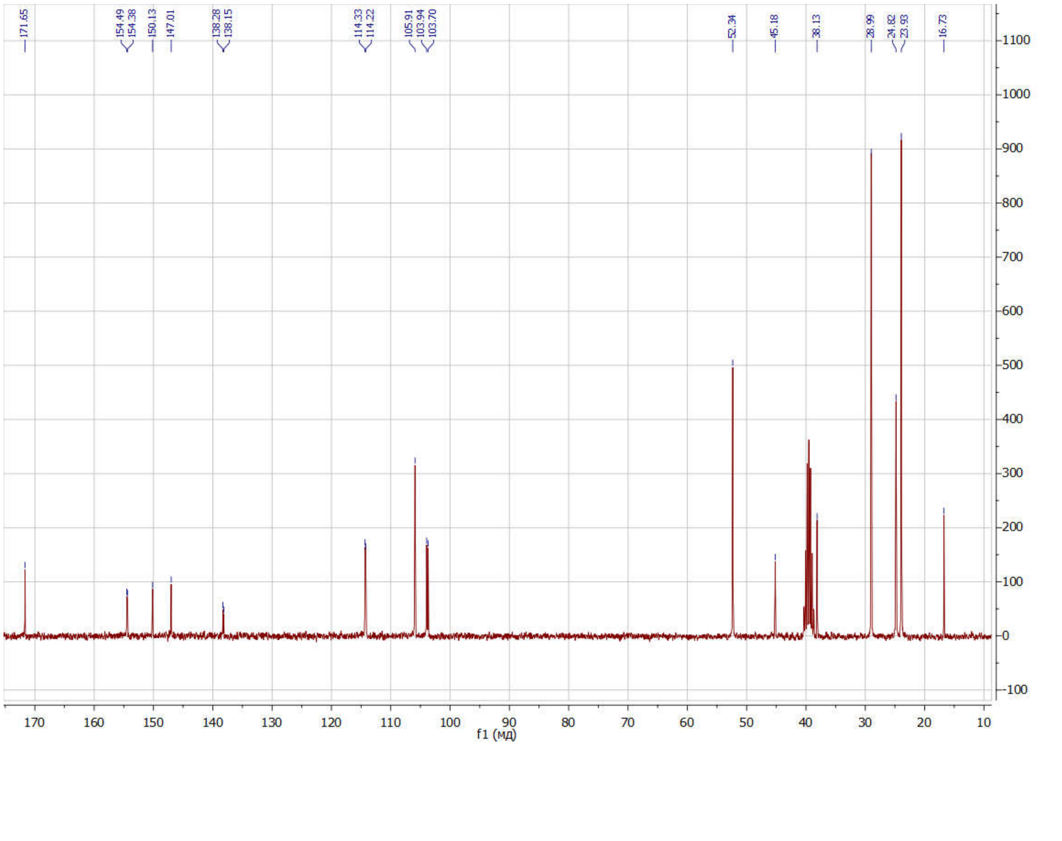

^{29}Si NMR spectrum of compound **7.**

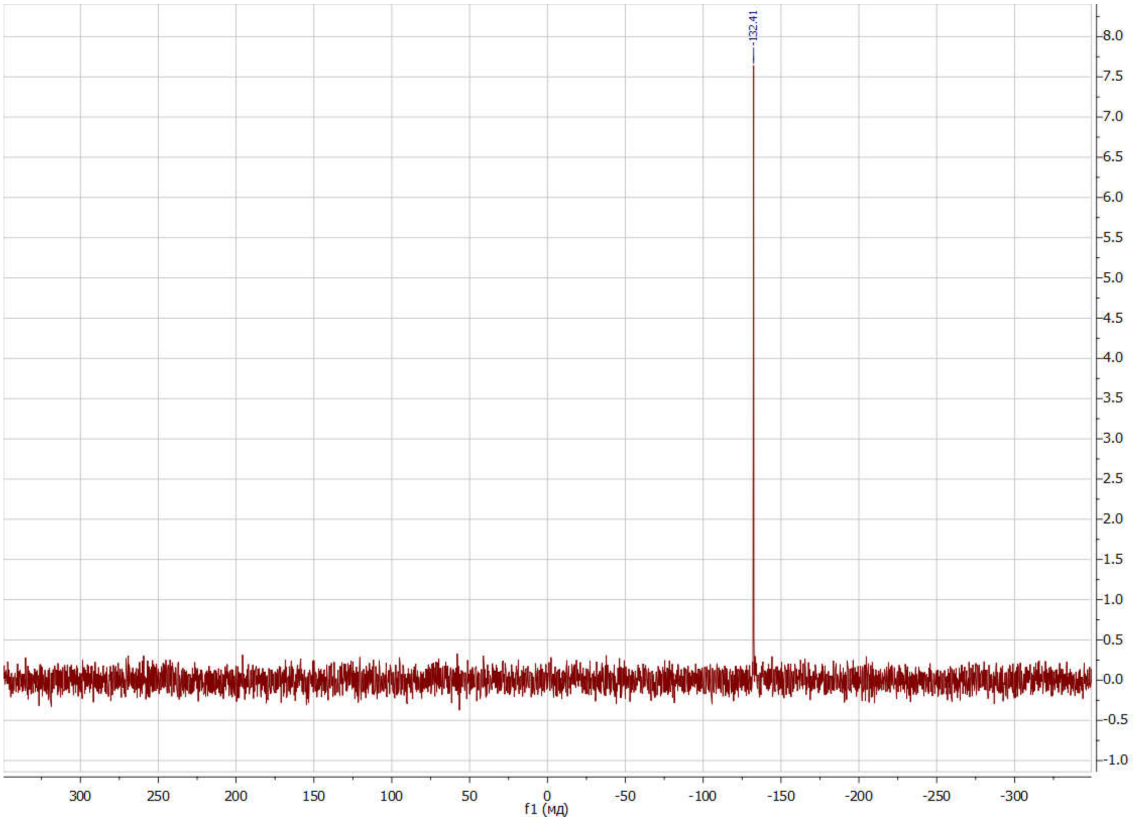

HRMS spectrum of compound **7.**

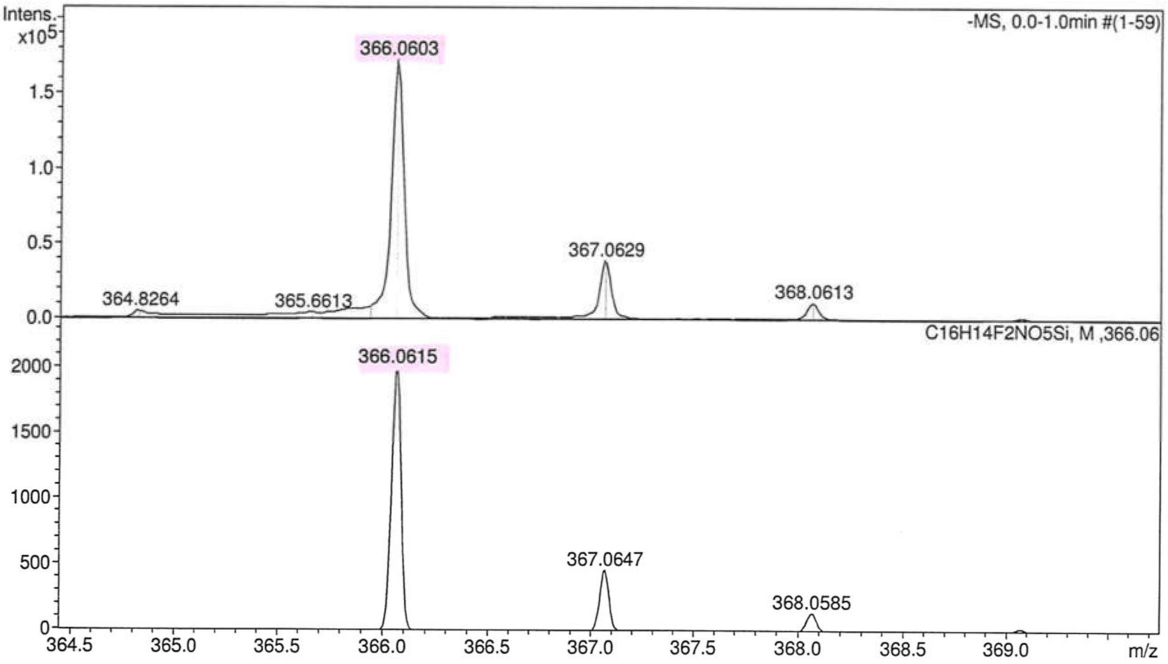

Supplement: Supplementary file 1 [file molecules-26-03548-s001.zip › molecules-1243982-supplementary.pdf]
